# A central role for PINK1 in governing local mitochondrial biogenesis and degradation in neurons

**DOI:** 10.1007/s00018-025-06054-4

**Published:** 2026-01-12

**Authors:** Marlena Helms, Angelika B. Harbauer

**Affiliations:** 1 TUM Graduate Center for Medicine and Health, Technical University of Munich, Munich, Germany; 2 Max Planck Institute for Biological Intelligence, Martinsried, Germany; 3 School of Medicine and Health, Institute of Neuronal Cell Biology, Technical University of Munich, Munich, Germany; 4 Munich Cluster for Systems Neurology, Munich, Germany

**Keywords:** Mitophagy, mRNA transport, Local translation, Mitochondrial proteases

## Abstract

Neurons have adapted the transport and positioning of mitochondria to fit their extended shape and high energy needs. To sustain mitochondrial function, neurons developed systems that allow local biogenesis and adaption to locally regulate mitochondrial form and function. Likewise, fine-tuned degradative systems are required to protect the neurons from mitochondrial dysfunction. Throughout both domains of mitostasis, the local synthesis of the mitochondrial damage-induced kinase PINK1 emerges as a central player. Along with other nuclear encoded mitochondrial proteins, its mRNA associates with mitochondria to sustain mitochondrial function locally. It also regulates mitochondrial degradation, via regulation of proteases, the generation of mitochondria-derived vesicles and mitophagy. In this review, we provide a general overview of the mechanisms governing mitochondrial health in neurons, with a special focus on the role of PINK1 in this endeavor.

## Mitochondrial dynamics in neurons

Mitochondria arose from an ancestral bacterium that was retained after engulfment by the precursor of eukaryotic cells, creating an endosymbiotic relationship that enabled the development of metazoans [1]. Reminiscent of their evolutionary origin, mitochondria resemble rod-like bacteria, especially in the axon of neurons where they mostly occur as solitary, roughly 1 µm long organelles [2]. In dendrites however, they can display a more elongated, fused morphology. Interestingly, even though mitochondrial density is high in dendrites, they form stable compartments that are isolated from each other as seen by the spread of a photoconvertible matrix protein [3]. Finally, in the soma of neurons, mitochondria form a reticular network [4]. How the different shapes of mitochondria in the neuronal sub-compartments are created or maintained is still incompletely understood.

Mitochondrial fission is regulated by recruitment of the cytosolic GTPase dynamin-related protein 1 (DRP1) to the mitochondrial outer membrane by various adaptor proteins, including mitochondrial fission factor (MFF) [5]. Fusion on the other hand relies on the two GTPases optic atrophy 1 (OPA1) in the inner mitochondrial membrane (IMM, [6]) and Mitofusin1/2 (MFN1/2) in the outer mitochondrial membrane (OMM, [7]). Unsurprisingly, depletion of MFF increases mitochondrial length, which is especially evident in axonal mitochondria, yet does not alter the mitochondrial mass within axons [8]. This suggests that the transport of mitochondria and their distribution throughout the axon follows some still undetermined rules that ensure proper occupancy. One study suggests that spacing of axonal mitochondria is determined by the local ATP supply, as removal of one mitochondrion by light-triggered activation of the phototoxic protein KillerRed targeted to mitochondria (mitoKillerRed) elicited a decrease in motility of nearby mitochondria, essentially enhancing their arrest at the site of depletion [9]. This aligns with the observation that mitochondria are stationed at sites of high energy demand, including the presynapse, nodes of Ranvier or axonal growth cones [10–12]. However, while neurons rely mostly on mitochondrially-derived ATP as a whole [13], not all synapses contain resident mitochondria, and glycolysis or diffusion may be sufficient to provide ATP at these sites [14, 15]. Nevertheless, mitochondria also fundamentally alter the synaptic release probability due to their Ca^2+^ buffering activity [16, 17]. However, the factors that promote mitochondrial arrest at some synapses but not others remain elusive.

**Fig. 1:**
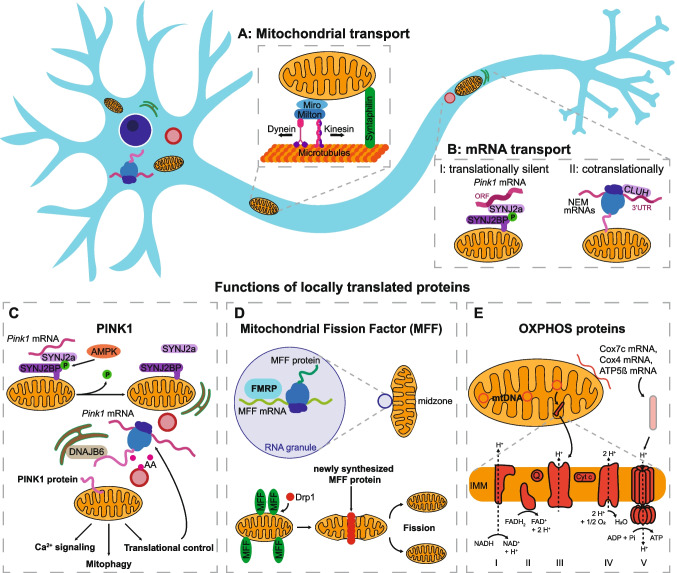
Biogenesis of mitochondrial proteins. **A** Mitochondrial transport along microtubules is facilitated by kinesin (anterograde transport) or dynein (retrograde transport) binding to Milton and Miro on the outer mitochondrial membrane, whereas Syntaphilin serves as a mitochondrial anchor. **B** Nuclear-encoded mitochondrial (NEM) mRNA can be transported along with mitochondria either translationally silent (I) or co-translationally (II). I: *Pink1* mRNA, following an initial translation step, is bound by SYNJ2a within a region of its open reading frame, preventing complete translation until the *Pink1* mRNA is eventually untethered. II: CLUH is able to bind NEM mRNAs through their 3’UTR, facilitating interaction with ribosomal subunits and co-translational transport of the mRNAs followed by import of the newly synthesized proteins into mitochondria. **C** The short-lived PINK1 protein is locally translated, following hitchhiking of its mRNA along with mitochondria via a complex consisting of SYNJ2a and its binding protein SYNJ2BP, an interaction that is enabled via SYNJ2BP phosphorylation by AMPK. Following local translation near endolysosomes, the PINK1 precursor is guided by the ER resident chaperon DNAJB6 towards mitochondria (ER-SURF pathway). Functions of the PINK1 protein include calcium signaling, mitophagy, or translational control. **D** The mRNA for MFF, on the other hand, colocalizes to mitochondria in the presence of FMRP within RNA granules, which are enriched at the midzone of mitochondria. MFF recruits Drp1 to the midzone of mitochondria and is thereby able to initiate mitochondrial fission. **E** For OXPHOS complexes, proteins from two different sources must be united: some of them are encoded within the mtDNA, allowing for local transcription as well as local translation. Others like Cox7c, Cox4 and ATP5ß are encoded in the nucleus and require transport of their mRNA along with mitochondria which allows them to be translated locally

Mitochondrial positioning has also been linked to the extension of axon branches [18], which is also negatively affected by MFF knockdown [8]. This may be due to the role that mitochondrially-derived ATP plays in supporting local translation in both axons and dendrites [3, 19], as local translation of cytoskeletal elements may be necessary for branch formation as well as for spine outgrowth. Vice versa, formation of actin cages around mitochondria serves to arrest mitochondria after their long-range transport on microtubules. Pharmacological depolymerization of Actin destabilizes the dendritic mitochondrial compartments [3] and mobilizes previously stationary mitochondria in the axon [20]. In addition, several mechanisms exist that regulate transport and arrest of mitochondrial transport along microtubules (reviewed by Pekkurnaz & Wang [21]). These often target the mitochondrial motor adaptor complex, consisting of the OMM protein RHOT1/2 (Miro1/2) and TRAK1/2 (Milton) [22–24], which connect mitochondria to kinesin and dynein, or the anchoring protein Syntaphilin [25], (Fig. 1A). Mitochondria move at a speed of approx. 0.5 µm/s in neurons with frequent pauses, and at any given time only a small fraction of mitochondria is observed in motion [26]. Their motility as well as their shape changes during development and aging, with motility decreasing as more and more mitochondria reach their final destination. As protein synthesis in neurons occurs primarily in the somato-dendritic area [27], this might lead to an aging population of mitochondria in distal axons over time. Indeed, a gradient of younger to older mitochondria along neurites is observed by the use of a mitochondrially-targeted fluorescent protein whose maturation from a protein emitting green fluorescence to red fluorescence has been engineered to occur only after approximately 24 h (mitoTimer), allowing a ratiometric readout of its relative age [28]. This probe however does not replicate the intricate relationship some mRNAs encoding mitochondrial proteins have with their encoded protein’s target organelle (see next chapter), and thus is not fully representative of the age of mitochondria.

Proper balance of mitochondrial dynamics is crucial for neuronal development and health. Following early differentiation, neurons undergo a switch in their metabolism from generating most of their energy via glycolysis in the beginning, to heavily relying on oxidative phosphorylation, and therefore mitochondria, for their energy production as mature neurons [13, 29, 30]. This metabolic switch is accompanied by changes in mitochondrial dynamics, involving both fusion and fission [31, 32], and also impacts neuronal size and complexity [33]. Neuron-specific knock outs of Drp1 in mice led to smaller forebrains, and primary cultures derived from these mice displayed less neurites, lower expression of synapse markers and disturbed mitochondrial distribution [32]. Loss of fusion on the other hand also impairs neuronal function. Purkinje cells in Mfn2-deficient mice decrease in size and display a decreased number of branches and spines. This was accompanied by a reduction in OXPHOS activity, in line with mitochondrial fusion being a prerequisite for maintenance of mtDNA [34]. Furthermore, mutations in Mfn2 have been linked to the Charcot-Marie-Tooth type 2 A (CMT2A) disease [35, 36]. One study suggested that Mfn2 can interact with Miro and Milton, and that an impaired Mfn2 could thus result in impaired axonal mitochondrial transport, possibly contributing to axonal degeneration in CMT [37]. Mitochondrial dynamics thus set the stage for proper mitochondrial distribution and function in the nervous system.

## Biogenesis of mitochondrial proteins in neurons is sustained by mitochondrial mRNA association

Mammalian mitochondria contain around 1500 proteins, of which more than 99% are encoded in the nucleus ([38–40]). In order to sort the nuclear encoded mitochondrial (NEM) proteins to the correct compartment after their synthesis on cytosolic ribosomes, specialized import pathways have evolved to accommodate the various topologies of proteins across the two mitochondrial membranes, the inter membrane space and the innermost matrix [41]. The translocase of the OMM (TOM complex) hereby serves as the main entry gate into the organelle and is equipped with receptors that recognize either a N-terminal amphipathic helix, the classical mitochondrial targeting sequence (MTS) recognized by Tom22/Tom20 [42], or internal sequences of mitochondrial protein precursors, that are guided by chaperones and recognized by Tom70 [43]. Chaperones are also crucial for mitochondrial protein import to prevent premature folding of mitochondrial precursors, as import needs to occur in an unfolded state to allow threading through the narrow tunnels of the translocases [44].

While most protein import into mitochondria can occur post-translationally, co-translational targeting has been observed, although it had been viewed as the exception, and can be enhanced by localizing translation close to the mitochondrial surface [45, 46]. Analysis of isolated mitochondria and proximity biotinylation approaches have identified a great wealth of mRNAs associated with mitochondria in a translation- dependent manner in various settings, ranging from yeast and plants to Drosophila and cultured human cell lines [47–49]. It has been reasoned that in fast growing organisms such as yeast, the speed of import may have to exceed the speed of protein translation at the ribosome in order to double the mitochondrial mass within one cell cycle, making co-translational import evolutionary unfavorable [50]. This may however not be as critical in slower dividing mammalian cells, and even less problematic in mature, postmitotic neurons. Indeed, two recent studies employed ribosome profiling on HEK293T cells and found that almost 20% of their identified mitochondrial proteins were co-translationally imported. Interestingly, the authors also observed that specifically proteins with a large size and complex topology relied on co-translational import [51, 52], with the interaction of the nascent chain with the import complexes likely being the driving force for the association of the mRNA. Interestingly, another observation included a second class of shorter proteins (under 200 amino acids) that were preferentially co-translationally imported which was due to their mRNAs being tethered to the OMM by interaction of the RNA-binding protein La ribonucleoprotein domain family member 4 (LARP4) with A-kinase anchoring protein 1 (AKAP1) on the mitochondrial surface [52, 53]. Such association of the mRNA enables the hitch-hiking of the transcripts along with mitochondrial trafficking. In neurons this mode of mRNA transport coupled to local translation is emerging as a fundamental mechanism to allow local repair and adaption of mitochondria in the distal parts of a neuron.

Generally, mitochondrial mRNA association is enhanced by RNA-binding proteins that interact either with ribosomal subunits, or directly with NEM encoding mRNAs [46, 54]. One of these proteins is clustered mitochondria homolog (CLUH), whose role in the stability and mitochondrial targeting of NEM mRNAs is conserved from yeast to human neurons [47, 55–58]. CLUH not only binds to the 3’UTR of many NEM mRNAs, but it also interacts with factors that enhance the re-initiation of translation at the same mRNA [55] (Fig. 1B-II). Loss of CLUH in neurons depletes the axonal pool of NEM transcripts, yet without affecting the movement of the remaining RNA particles [55]. Two scenarios may explain this observation: (i) CLUH has been shown to affect the stability of its clients, thus the lower abundance in axons may simply be a consequence of the reduced half-life of NEM mRNAs. This matches data from global mRNA abundance measurements in cultured neurons, that find a relationship between the mRNA stability and the likelihood of its axonal localization [59]. (ii) As CLUH is necessary to localize ribosome recycling factors to the axon, it ensures the continued reassociation of the ribosomes to the same mRNA after completion of the first round of translation. This allows continued translation of the same NEM transcript and thus exposure of an MTS in close proximity to a potentially moving mitochondrion, and a continued interaction between the TOM complex and the MTS/ribosome/mRNA complex. In line with this idea, overexpression of the ribosome recycling factor ABCE1 rescues mRNA abundance and growth deficits in CLUH knockout motoneurons [55].

The mitochondrial hitch-hiking of mRNAs was experimentally shown to be the case for the *Pink1* mRNA [60], as well as for the *Cox7c* mRNA [61], using live cell imaging of mRNAs labelled by the MS2-tagging approach in cultured neurons. However, hitch-hiking of NEM encoding mRNAs is not restricted to mitochondria. In recent years, mRNAs which are important for mitochondrial functions have been shown to also depend on the transport of endosomes and endolysosomes for their axonal localization [62, 63]. Also other organelles, such as early endosomes, have been shown to associate with NEM encoding mRNAs [64]. Interestingly, several of these hitch-hiking events can be prevented by destabilization of the ribosome by treatment with Puromycin, suggesting that the nascent polypeptides may be involved in the targeting to the organelle. In the case of mitochondria, this can be mediated by an N-terminal MTS, as was shown both for *Pink1* and *Cox7c* [60, 61]. How the translation-dependent targeting would allow an association of NEM nascent proteins to organelles of the endolysosomal system remains to be determined, as the nascent chains of NEM proteins would not find suitable receptors on these types of membranes. In addition to organellar hitch-hiking, some NEM mRNAs without a classical MTS have been shown to be transported within an mRNA granule, containing the RNA binding protein SFPQ, and to be locally translated within the vicinity of mitochondria [65]. Likewise, RNA granules marked by the RNA binding protein FMRP colocalize with the mRNA for MFF [66], allowing its local translation.

Local translation is especially relevant for short-lived proteins like PINK1, as the time to travel to the distal parts of the neurons exceed its life-time [67–69]. Local translation of PINK1 therefore ensures its availability for the detection of dysfunctional mitochondrial also in distal parts of axons, as will be outlined in the mitophagy chapter. Fittingly, the association of the *Pink1* mRNA with mitochondria is not observed in fibroblasts unless its anchoring complex is overexpressed [60], as smaller (“shorter”) cells may not need to add this additional layer of regulation. Unlike *Cox7c*, *Pink1* mRNA association is not only driven by translation. After an initial, translation and MTS-dependent targeting to the OMM, the *Pink1* mRNA becomes tethered to the OMM by binding to Synaptojanin 2a (SYNJ2a) and its binding protein SYNJ2BP [70, 71] (Fig. 1B-I). SYNJ2a acts as the RNA binding protein in this complex, and interestingly binds within the coding region of the PINK1 open reading frame [60]. This suggests that unlike most NEM transcripts, the *Pink1* mRNA may be transported in a translationally silent state and needs to be untethered from its mitochondrial association to allow access of the ribosome to the part of the ORF that is otherwise bound by SYNJ2a.

## Local translation of mitochondrial proteins occurs at organellar contact sites

Organellar hitch-hiking not only plays an important role in the transport of mRNAs into the axon, but also may be responsible for the localization of ribosomes within the axon. Ribosomes in axons, while rare and mostly translating as monosomes [72], seem to preferentially associate with endomembranes, including the ER [73] or early endosomes via the FERRY complex [64]. Removal of the ER from axons by heterodimer-induced forced association of retrogradely moving motor proteins overall reduced axonal protein synthesis. A similar effect was observed for the knock down of p180/RRBP1, a ribosomal receptor on the ER [73]. Unlike the other ribosomal receptor on the ER, Sec61, RRBP1 is not directly associated with the ER translocon, which allows ER-associated protein synthesis to exist uncoupled from import of the nascent chain into or across the ER membrane [74]. This would allow the synthesis of not only secretory proteins targeted to the ER, but also ER-associated synthesis of cytosolic or even mitochondrial proteins. Whether the ER-associated ribosomes are actively transported into the axon along with ER tubule dynamics or whether they arrive in the axonal compartment by lateral diffusion or other means of transport has not been addressed experimentally. In contrast, association of ribosomes to early endosomes has been shown to depend on components of the FERRY complex [64]. This suggests that hitch-hiking on early endosome during their transport into the axon may provide an active localization mechanism for axonal ribosomes.

Fittingly, it was shown that local hotspots of translation form at contact sites between mitochondria and endolysosomes, including translation of the OMM protein VDAC2 [62]. This is also the case for the local translation of MFF [66], where ribosomes at these contact sites were also visualized by CryoET. In the case of PINK1, local translation at mitochondria endolysosome contact sites was not only observed in axons but also in the soma [75]. Interestingly, the translation of PINK1 at these sites is regulated by metabolic signaling as association of the *Pink1* mRNA to mitochondria depends on phosphorylation of SYNJ2BP by AMP-activated kinase (AMPK), which stabilizes the interaction between the RNA-binding protein SYNJ2a and the OMM protein SYNJ2BP [76]. Interestingly, this association limits the translation and subsequent functionality of PINK1. Inhibition of AMPK, as it occurs downstream of insulin signaling due to inhibitory phosphorylation of AMPK by AKT, leads to the dissociation of the *Pink1* mRNA and its subsequent localization near endolysosomes (Fig. 1C) [76]. However, as a mitochondrial protein, the newly synthesized PINK1 precursor needs to find its way back to mitochondria. Using correlative light and electron microscopy in combination with a PINK1 translation reporter, we now suggest that the gap between endolysosomes and mitochondria may be filled by the ER [75] (Fig. 1C). This enables the transport of the precursor of this transmembrane protein along the ER surface in order to reach mitochondria in association with ER-bound chaperones like DNAJB6 [75], as was shown for other mitochondrial transmembrane proteins in yeast [77]. This fits well with the above described role for ER-associated ribosomes in local protein synthesis.

On the other hand, the role of the endolysosomes in local PINK1 protein synthesis is less clear. While early endosomes may bring in the required ribosomes for PINK1 synthesis via the FERRY, the PINK1 protein translation sensor rather colocalized with markers of late endosomes or lysosomes [75]. Maturation of FERRY-positive endosomes into late endolysosomes may underlie this observation, but this hypothesis needs to be tested. Additionally, the lysosomal surface serves as a signaling hub for both mTORC1 and AMPK signaling, and thus formation of mitochondria lysosome contacts may elicit the untethering of the *Pink1* mRNA from SYNJ2BP on mitochondria. However, inhibition of mTORC1 does not prevent the untethering of the *Pink1* mRNA [76], indicating that while activation of mTORC1 may contribute to the increased biogenesis of PINK1 upon activation of insulin signaling due to its effect on general translation initiation factors, it is not necessary for the initial untethering event. Finally, lysosomes will produce a local supply of amino acids depending on their degradative capacity. While this has not yet been shown to matter for local translation in neurons, a preprint observes a similar effect of lysosomes on translation at three-way junctions of ER in cultured cell lines [78]. Thus, lysosomes may serve as amid acid reservoirs across cell types.

## Functions of locally translated mitochondrial proteins

### Mitophagy, Ca2+ homeostasis and translational control exerted by PINK1

The best-known function of PINK1 is the detection of damaged mitochondria and their demarcation for mitophagy, as will be described in the next chapter. Local translation of PINK1 therefore ensures the continued supply of this protein also to distal mitochondria in both axons and dendrites [60].

Beyond mitophagy, activation of PINK1 in response to mitochondrial damage has been described to repair rather than remove damaged mitochondria. It was shown in Drosophila neurons as well as in HeLa cells that PINK1 overexpression, but not expression of a Parkinson’s disease (PD)-linked mutant, leads to stimulation of localized translation of mRNAs encoding subunits of the respiratory chain [79]. This is achieved via phosphorylation and subsequent proteasomal degradation of translational repressors, including Pumilio and Glorund/hnRNP-F [79]. Thus, the local translation of PINK1 at endolysosome-mitochondria contact sites will lead to a de-repression of translation and favor the local translation of also other mitochondrially-associated transcripts in a positive feedback loop (Fig. 1C). However, in Drosophila oocytes, PINK1 activation also prevents the transmission of deleterious mtDNA mutations through the germline by limiting the local production of factors necessary for mtDNA replication [80]. To achieve this, PINK1 activation on dysfunctional mitochondria leads to the phosphorylation of the RNA binding protein Larp, which is bound to the OMM protein MDI and normally mediates the localized translation of NEM mRNAs at the OMM [80, 81]. This in turn dampens translation of mitochondrially-associated mRNAs [80]. Thus, PINK1 activation can have opposite effects on localized translation near the OMM depending on the model system used. In mammalian cells, the Larp homologue LARP4 also binds nuclear-encoded mitochondrial transcripts [82], as does the MDI homologue AKAP1 [53], yet whether PINK1 plays a more direct role in coordinating localized translation in mammalian neurons remains to be determined.

Another role of PINK1 includes effects on local Ca^2+^ uptake and release from mitochondria, either directly via phosphorylation of LETM1, a putative Ca^2+^/H^+^ antiporter in the IMM [83], or indirectly via inhibition of PKA-mediated phosphorylation of mitochondrial Na^+^/Ca^2+^ exchanger, NCLX [84]. Indeed, the major phenotype of PINK1 loss in neurons is not the accumulation of damaged mitochondria, but an increase in the cytoplasmic Ca^2+^ concentration that leads to cell death [85]. How local translation of PINK1 affects the ability of individual mitochondria to modulate the local Ca^2+^ flux will be an interesting field of study. Whether any of the other regulators of mitochondrial Ca^2+^ flux are locally translated has not been studied. However, given the idea that modulation of Ca^2+^ flux may be the main function of mitochondria in the axon, this would be a powerful way to further tune synaptic signaling through local protein translation.

### Mitochondrial fission induced by FMRP mediates local translation of MFF

The RNA binding protein FMRP was recently shown to localize to sites of mitochondrial fission in neurons [66]. These fission events were characterized by their symmetrical nature, which is attributed to MFF-mediated recruitment of DRP1 [86]. Indeed, FMRP-granules colocalized with MFF mRNA and local translation of MFF could be observed at mitochondria-endolysosomal contact sites ([66], Fig. 1D), in agreement with the notion that these organellar contact sites serve as translational hotspots in neurons [62]. This may also explain the previous observation that endolysosomes mark fission events in neurons [87]. The presence of ribosomes at these contacts was corroborated by Cryo-electron microscopy, along with ER tubules marking the fission site [66]. This association of fission events with FMRP granules was not a frequent observation in non-neuronal cells, indicating that this mechanism may represent a unique adaption to the specific needs of neurons. In line with this, loss of FMRP leads to a reduction in MFF mRNA presence in axons, reduced association of the MFF transcript with mitochondria, and a reduction of mitochondrial fission in axons [66], suggesting that it impairs both the transport and the translation of the MFF transcript.

### Local translation of OXPHOS components and the question of mtDNA-encoded subunits

On a more global scale, quantitative mass spectrometry and in vitro stimulation of isolated mouse synapses revealed that the synthesis of mitochondrial proteins is upregulated in response to NMDA administration [88]. Many of these newly synthesized proteins comigrated with complexes of the respiratory chain in Blue Native PAGE, suggesting that they are assembled into functional complexes [88]. This fits with the mitochondrial hitch-hiking of the mRNA encoding Cox7c as a subunit of complex IV of the respiratory chain [61]. Additionally, there is evidence that other mRNAs encoding further subunits of complex IV and V also co-localize with mitochondria in neurons [60, 76, 89, 90]. While it has not been directly shown, this suggests that also the ability to perform OXPHOS may be altered by local translation of mitochondrial OXPHOS components (Fig. 1E). In favor of this hypothesis, addition of a protein translation inhibitor to the axonal compartment of neurons, cultured in compartmentalized chambers, decreases the membrane potential across the inner mitochondria membrane [91], which is generated by the respiratory chain complexes I, III and IV. However, it is unclear whether this represents exchange of e.g. short-lived peripheral subunits [92], or a concerted de novo biogenesis of completely new complexes. Recently, the turnover of OXPHOS complexes was measured by feeding mice a pulse of the stable nitrogen isotope ^15^N followed by mass spectrometric analysis of mitochondria of different tissues, including whole brain [93]. This revealed that some mitochondrial proteins in brain mitochondria, including many subunits of the respiratory chain complexes, display exceptionally long half-lives of up to several months. On average, complex III and V are more long lived than complexes I, II, and IV, and membrane-embedded subunits have higher stability than matrix-exposed subunits e.g. within complex I [93]. While this suggests that the matrix-exposed subunits may benefit from replacement via local translation of their encoding mRNA in the long run, it remains doubtful whether this would be measurable in the short timeframe stimulation with NMDA or the inhibition of axonal translation. This leaves the possibility that de novo biogenesis of OXPHOS complexes may occur locally.

However, de novo biogenesis of most respiratory chain complexes also requires the incorporation of one or more proteins that are encoded within the mitochondrial genome (mtDNA, Fig. 1E). The coordination between cytoplasmic translation with the translation of mtDNA to generate stochiometric amounts of proteins is an ongoing area of research even in non-neuronal cells [94]. To complicate the matter, it has been suggested that some, if not most, axonal mitochondria lack mtDNA [95], questioning the idea that de novo synthesis of complete respiratory chain complexes can occur in axons. Indeed, the majority of electron transport chain complexes seemed to be downregulated in synaptic mitochondria [96]. This is in line with results from a recent preprint, where the authors performed proteomics on mitochondria isolated from MitoTag mice and found that axonal mitochondria possessed reduced mtDNA expression levels. In addition, compared to their somato-dendritic counterparts, axonal mitochondria showed decreased levels of proteins involved in translation and oxidative phosphorylation, and instead seemed to favor fatty acid oxidation [97].

There is however evidence that local translation of mitochondrially encoded proteins actually happens in axons: Using clickable non-canonical amino acids in the presence of inhibitors of the cytoplasmic ribosome, mitochondrial translation was detected in both axons and dendrites of neurons in culture [98]. Additionally, local translation of mitochondrial initiation factor 3 (mtIF3) has been reported in axons in response to brain-derived neurotrophic factor (BDNF) signaling [99]. This is one of only two translation initiation factors in mitochondria, and fittingly, its local translation boosts formation of the mitoribosome in axons [99]. Two possible solutions could reconciliate the absence of mtDNA with continued translation of mitochondrially encoded mRNAs. Firstly, mitochondria that enter the axon lacking mtDNA may still carry enough mRNA to sustain a limited amount of de novo biogenesis of OXPHOS complexes in the periphery. As also the mRNA associated to the outside of mitochondria cannot be replenished, this scenario seems reasonable, yet also places a “best before” date on an individual mitochondrion, unless it is resupplied by fusing with a younger mitochondrion or replaced altogether. How frequently mitochondria are replaced in the periphery is a matter of debate, as with increasing age and maturity of neurons, less and less mitochondrial transport is observed in cortical axons in vivo [100]. However, this may also vary by the cell type, as a similar reduction has not been observed in axons of retinal ganglion cells even in aged mice [101]. How different cell types manage their mitochondrial biogenesis and whether they all rely to the same extent on mitochondrial transport and local translation of mRNAs encoding mitochondrial proteins remains an active area of research.

The second solution takes advantage of the fact that not all mitochondria entering the axon lack mtDNA. Even in the most extreme examples, around 10% of axonal mitochondria still carried mtDNA [95]. It may be exactly these mitochondria that serve as a local center of OXPHOS biogenesis. This is in line with the idea that translational hotspots occur not at all mitochondria equally, but may be allocated to mitochondria in specific positions, such as at axonal branch sites or in direct contact with an endolysosome. However, it remains an open question how this localized de novo biogenesis of OXPHOS complexes would benefit mitochondria outside of these specialized positions. Mixing of mitochondrial content may occur over time through fission and fusion, which are however restricted in axons due to the high number of stationary mitochondria [26]. Nevertheless, mtDNA replication also occurs in axons [102] and is associated with MFF driven fission [66], which could increase the number of “seeds” for de novo OXPHOS complexes. Replication of mtDNA depends on the translation-elongation factor eEF1A1 of cytoplasmatic translation, suggesting coordination between cytoplasmic translation and the replication of mtDNA [102] that may also occur at the translational hotspots within the axon.

## Degradation of mitochondrial proteins in neurons

**Fig. 2:**
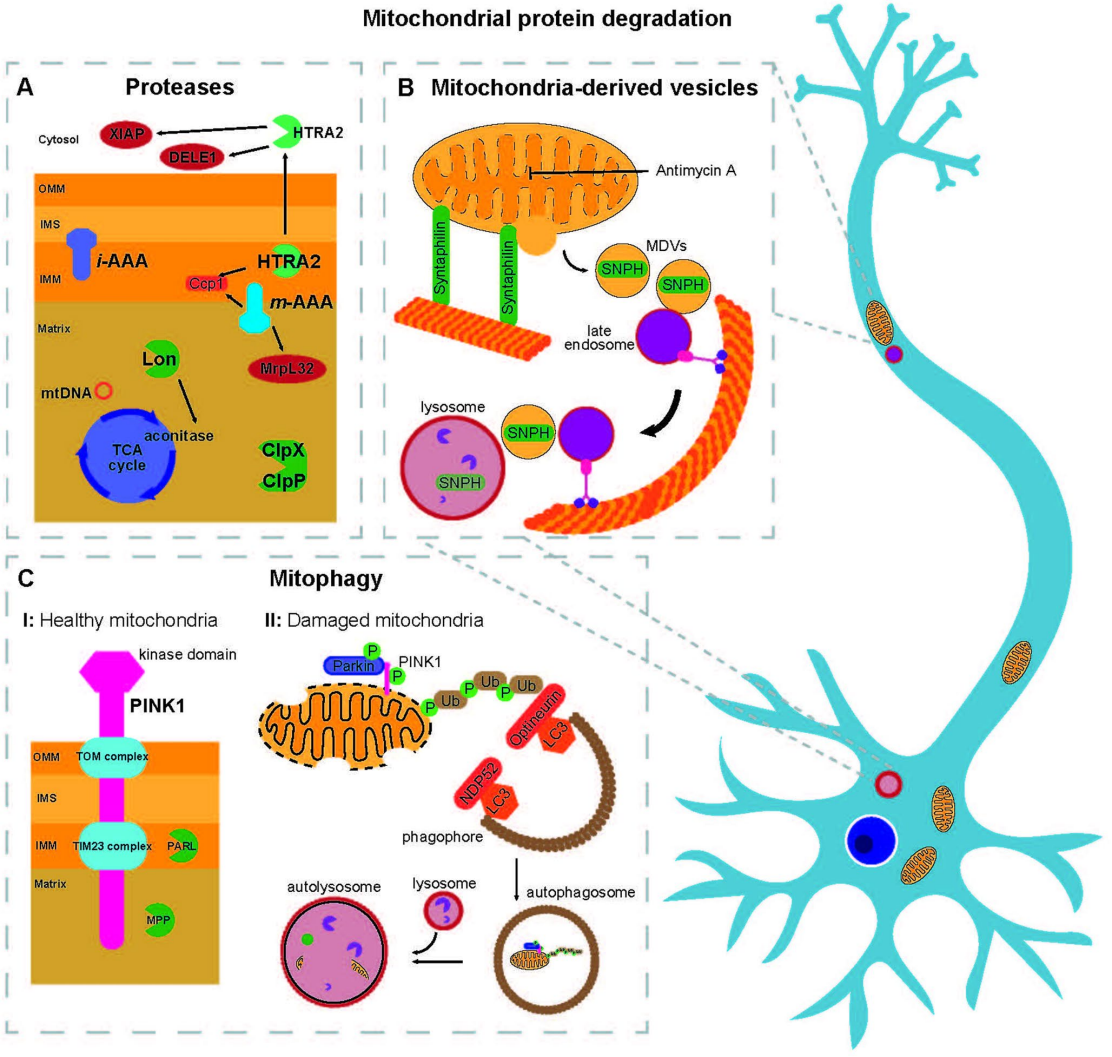
Mitochondrial protein degradation. **A** Mitochondrial proteases can be divided based on their localization: ClpPX and Lon make up the proteases surveilling the mitochondrial matrix, with Lon targeting both mitochondrial DNA as well as aconitase, an enzyme of the TCA cycle. The inner mitochondrial membrane (IMM) contains the *i*-AAA, *m*-AAA (targeting the cytochrome c peroxidase (Ccp1) as well as the ribosomal protein MrpL32), and HTRA2. HTRA2 is able to process itself and subsequently translocates to the cytosol where it processes its targets XIAP and DELE1. **B** In healthy mitochondria, PINK1 is quickly imported and processed by the proteases PARL and MPP (I). In contrast, when mitochondria are damaged and their membrane potential is depolarized (II), PINK1 stabilizes on the mitochondrial surface where it then phosphorylates target proteins, resulting in the recruitment of Parkin to mitochondria, which in turn ubiquitinylates phosphorylated proteins. This results in a positive feedback loop leading to the formation of phospho-ubiquitin chains, which can be recognized by NDP52 or Optineurin, and subsequently LC3, triggering the formation of a phagophore membrane around the tagged mitochondria. Upon fusion with lysosomes, autolysosomes are formed and digest the damaged mitochondria. **C** Mitochondria-derived vesicles (MDV) can form upon mitochondrial damage, for example triggered by treatment with Antimycin A, and package specific cargo, e.g. Syntaphilin (SNPH). SNPH-containing MDVs can then be transported along with late endosomes towards the soma of neurons where they are degraded by lysosomes. Additionally, treatment with Antimycin A can result in the formation of MDVs in a Parkin/PINK1-dependent manner that results in lysosomal degradation of these MDVs independently of mitophagy as described in B.

In order to ensure mitochondrial quality control, different approaches can be taken by the cell. First, mitochondria possess their own set of proteases and chaperones, aiding in the processing and proper folding of mitochondrial proteins [103, 104]. Secondly, damaged mitochondria can be removed via a mitochondria-specific form of autophagy – mitophagy [105]. In recent years, mitochondria-derived vesicles (MDVs) have also been investigated as a mitochondrial quality control mechanism [106] (Fig. 2).

## Proteases

The electron transport chain residing in the IMM is a major source of reactive oxygen species within the cell [107], which easily oxidize proteins within mitochondria, often resulting in their inactivation [108]. This is counteracted by a system of proteases within mitochondria (Fig. 2A) that not only keep mitochondrial homeostasis by removing misfolded or oxidized proteins, but also play important regulatory functions during protein import and complex assembly (reviewed by Deshwal et al. [107]).

All inner-mitochondrial compartments contain dedicated proteases for mitochondrial protein quality control: Lon and CLpXP (caseinolytic peptidase P) proteases are located within the matrix [109–111], while two proteases of the ATPases Associated with various cellular activities (AAA) family surveil the inner membrane with one facing the matrix (*m*-AAA) and the other facing the intermembrane space (IMS, *i*-AAA) [112–114]. Additionally, the IMS is also guarded by the HTRA2 (high temperature requirement A2)/Omi protease [115]. As they are located within mitochondria, it is assumed that they will be present in all neuronal subcompartments, but experimental evidence for this is still lacking. However, their importance in neurons is evident due to the link between genetic mutations in some protease subunits and neurodegenerative disorders. For example, HTRA2 mutations are linked to PD [116, 117] and deletions as well as mutations of Paraplegin, an *m*-AAA subunit, have been associated with hereditary spastic paraplegia (HSP) [118, 119], an upper motor neuron disease. Thus, mitochondrial proteases form the first layer of defense against mitochondrial dysfunction in neurons.

In the matrix, an oxidized form of the TCA cycle enzyme aconitase is degraded preferentially by the Lon protease [120]. In line with this, downregulation of Lon leads to the accumulation of damaged aconitase [120], as well as to reduced OXPHOS assembly and even cell death [121]. Lon also binds to mitochondrial DNA directly and associates with Twinkle, the helicase for mtDNA [122, 123]. Lon is therefore not only an important part of the degradative system of mitochondria, but may also regulate mitochondrial protein biogenesis through its processing of proteins responsible for mtDNA replication or transcription [123]. In neurons, Lon protease was investigated in the context of the PD model of treatment with the toxin MPTP (1-Methyl-4-phenyl-1,2,3,6-tetrahydropyridin), which selectively damages dopaminergic neurons due to its conversion into the neurotoxin MPP^+^ [124, 125]. This leads to an accumulation of oxidized and carbonylated proteins, including aconitase and OXPHOS proteins, consistent with a loss of Lon function [125]. Although human post-mortem tissue of PD tissue revealed increased expression levels of Lon, the protease remained inactive, possibly contributing to the accumulation of damaged proteins [125]. How MPP^+^ causes Lon dysfunction remains unclear, yet this study demonstrates the tight link between mitochondrial quality control and PD beyond the genetic linkages mentioned above. Enhancing protease function in PD might therefore be an interesting avenue for future research.

Similarily, Clp protease can counteract PD-associated phenotypes [126]. In eukaryotes, the protease named ClpXP is made up of two components: the AAA + ATPase ClpX, which unfolds protein targets, and the peptidase ClpP, which is responsible for the protein degradation [127]. Decrease of ClpP in dopaminergic SH-SY5Y cells is triggered by accumulation of the PD-associated protein aggregate alpha-synuclein and leads to the increased production of reactive oxygen species [128]. Conversely, enhancing ClpP levels in alpha-synuclein A53T mice, a PD model carrying a missense alpha-synuclein mutant, reduces the pathological phosphorylation of alpha-Synuclein at serine 129 [126].

Moving from the matrix to the mitochondrial inner membrane, the *m*-AAA consists of a complex containing paraplegin and AFG3L2, but AFG3L2 is also able to form a homo-oligomeric complex [129–131]. In the brain, Afg1l1 is another, less abundant, complex component of the *m*-AAA [131]. Targets of the *m*-AAA include cytochrome c peroxidase (CCp1), and the ribosomal mitochondrial protein MrpL32 [131]. Failure to process the latter protein has been associated with impaired mitochondrial protein synthesis [132], again linking degradative function to protein biogenesis in mitochondria. Paraplegin-deficient mice were also characterized by neurobiologically defects such as impaired axonal transport, visually affected mitochondria (e.g. abnormal cristae), and progressive axonal degeneration [119].

HTRA2/Omi is another protease shown to localize to the IMM under normal conditions, but is able to catalytically process itself before it translocates to the cytosol. There it is able to interact with its targets, including the X-linked inhibitor of apoptosis proteins (XIAP), which is inhibited by this interaction [133–135]. Downstream, this interaction has been shown to result in cell death [133]. However, HTRA2/Omi appears to serve a neuroprotective role as knockdown of the protease in mice resulted in abnormal neurological behavior, neuron loss and early lethality [134], fitting to its association with PD [136]. Recently, DELE1, a sensor for unfolded proteins within the mitochondrial matrix (UPR_mt_) [137, 138], has been reported to be another substrate of HTRA2/Omi [139]. Cleaved DELE1 binds and stimulates HRI [137], which in turn phosphorylates eIF2alpha and initiates the integrated stress response (ISR) [140]. Activation of the ISR attenuates cytoplasmic translation, but enables the specific translation of the transcription factor ATF4 [141]. This may also occur in the periphery of neurons, as also the mRNA of ATF4 is locally available [142] and, like other locally synthesized nuclear proteins [143], its retrograde transport will enable transcriptional changes triggered by the ISR. However, whether the transcriptional response, including the upregulation of mitochondrial proteases and chaperons, can in any way be targeted to the source or if all mitochondria will benefit from the change is unknown. Depending on the cell line, DELE1 can also be cleaved by another mitochondrial protease, OMA1 [144]. In fact, loss of HTRA2 was linked to increased instead of decreased induction of CHOP [145], an ATF4 target, suggesting that OMA1 may compensate for the loss of HTRA2 and mediate DELE1 cleavage. However, the absence of HTRA2 still increased levels of ROS and caused an excess of unfolded proteins in mitochondria [145], supporting its role in ameliorating UPR_mt_.

Interestingly, HTRA2/Omi itself seems to be phosphorylated by PINK1 upon mitochondrial stress, likely resulting in its enhanced activity. Subsequently, PD patients carrying PINK1 mutations show decreased HTRA2 phosphorylation levels in brain tissue [136]. It would be tempting to speculate that the stress response enhancing HTRA2 phosphorylation by PINK1 is also linked to mitophagy or an altered UPR_mt_, but so far, the involvement of HTRA2 in the PINK1/Parkin pathway of mitophagy has been disputed [146, 147].

## Mitophagy

Mitochondria can be subjected to a special form of autophagy, called mitophagy [105]. Selective, distinct pathways cull mitochondria upon different cues, such as during the elimination of mitochondria during erythrocyte development, during hypoxia or upon mitochondrial damage [148]. Some basal turnover of mitochondria is required in neurons to balance mitochondrial biogenesis to maintain mitochondrial numbers [26]. Overall, mitophagic flux in neurons is rather low [149], which may be due to a high expression of negative regulators of autophagy [150], which restricts also the degradation of mitochondria by general, non-selective autophagy. Interestingly, mitochondrially-derived proteins, especially those associated with mtDNA, still make up a major factor of the autophagosomal content in the brain [151], arguing that also targeted mechanisms to remove mitochondria or mitochondrial content contribute to the turnover of mitochondria in the brain. Some autophagosomes form at the tip of axons and mature on their way to the soma [152], while other studies suggest that mitochondria first move retrogradely before they are captured by the autophagosomal machinery [153, 154] or are exclusively degraded in the soma [155]. To reunite all these different models, it will be imperative to understand which pathways trigger basal mitophagy in neurons. Selective basal mitophagy has been hypothesized to be independent of the damage-induced PINK1-Parkin pathway (see below) [149] and instead to be mediated by receptors such as the Bcl-2 interacting protein 3 (BNIP3L). BNIP3L has been shown to be responsible for mitophagic flux during neuronal development [156], but also partially appears to be able to compensate for PINK1 deficiency in more mature neurons [157]. The mechanisms of BNIP3L-mediated mitophagy are reviewed elsewhere [158]. It is likely that both redundant mechanisms for basal mitophagy in different subcellular compartments as well as dedicated mechanisms to detect damaged mitochondria coexist, and that neuronal cell type diversity is reflected in their reliance on one pathway over the other.

Most work in neurons has focused on the damage-induced PINK1-Parkin pathway of mitophagy (Fig. 2B), given its association to PD [159, 160]. As a mitochondrial protein, PINK1 resides on mitochondria, orienting its C-terminal kinase domain towards the cytoplasm [161]. In healthy cells with an intact mitochondrial membrane potential, PINK1 is quickly imported into mitochondria via TOM and TIM23 [162, 163] before it is primarily cleaved by PARL and MPP, but also by *m*-AAA and ClpXP [164, 165], and protein remains undergo proteasomal degradation [166].

However, when the mitochondrial membrane potential is disrupted, PINK1 no longer gets imported into mitochondria and instead stabilizes on the OMM [167, 168], where it subsequently undergoes autophosphorylation [169] and phosphorylates target proteins such as ubiquitin [170–172]. Another one of these target proteins is Miro, resulting in its degradation and subsequently halting mitochondrial movement [173].

Additionally, phosphorylation of ubiquitin in the vicinity of the OMM leads to the recruitment of the E3 ubiquitin ligase Parkin to mitochondria [169, 170, 174, 175]. Parkin continues to ubiquitinate phosphorylated OMM proteins [176], and these ubiquitin chains are then further phosphorylated by PINK1 [177]. PINK1 also phosphorylates Parkin at Serin 65, further enhancing its activity [171]. These reactions of phosphorylation and subsequent ubiquitination result in the formation of phospho-ubiquitin chains on the damaged mitochondria [177]. The first reaction to the phosphorylation of OMM proteins is in most cases their selective removal from the OMM and degradation via the proteasome [176]. This is the case for Miro [173] as well as the Mitofusins [178]. This results in mitochondrial fragmentation, preventing the fusion of damaged with healthy mitochondria, and thereby promoting mitophagy [179]. Recruitment of the AAA + ATPase, p97/VCP, to mitochondria helps with the extraction of the OMM proteins from the membrane and the release of mitochondria from ER contacts mediated by those OMM proteins [180].

Extensively phospho-ubiquitinated mitochondria can be recognized by autophagy receptors, including NDP52 and optineurin [181]. Proteins on phagophores, such as the microtubule-associated protein light chain 3B (LC3B) are also recruited, resulting in the formation of autophagosomes around the labelled mitochondria [182]. Fusion with lysosomes and the formation of autolysosomes is followed by the acidic degradation of the damaged mitochondria [182]. Interestingly, a recent pre-print suggests that while PINK1-Parkin activation can take place throughout the axon and its terminals, phagophore formation seemed to be spatially restricted to boutons, placing this step of mitophagy in the necessary lipid-rich environment. While this may be beneficial for facilitating mitophagy, it also appears to make pre-synapses more vulnerable to mitophagy-defects, again highlighting the importance of this pathway especially in neurons [157].

## Mitochondria-derived vesicles (MDVs)

While mitophagy clears whole mitochondria, the enrichment of some but not all mitochondrial proteins in autophagosomes in the brain suggests that piecemeal forms of autophagy must exist in the brain [151]. Also other pathways, including mitochondria-derived vesicles, could deliver selected cargo to lysosomes. Only the size of 60–150 nm, MDVs originate from mitochondria mostly independently of DRP1, and selectively choose their cargo [183, 184], before its delivery to lysosomes or peroxisomes ([185], reviewed by Sugiura et al. [104]. MDV transport can be initiated e.g. by oxidative stress, which increases their loading with oxidized subunits of complexes II, III and IV, and may contain both OMM and IMM membranes [184]. Knockdown of Parkin also resulted in less formation of an MDV-subtype upon Antimycin A treatment, a process that was also dependent on the presence of PINK1 [186] (Fig. 2C, upper part). Interestingly, this elimination of damaged proteins via MDVs could act as a mitochondrial quality control mechanism, preceding loss of the mitochondrial membrane potential and subsequent mitophagy [186].

In neurons treated with Antimycin A, Lin et al. [182] observed a reduction in Syntaphilin in axonal mitochondria. Immuno-electron microscopy revealed that Syntaphilin was indeed redistributed within stressed mitochondria and eventually shed via MDVs. However, these MDVs do not remain in the vicinity of mitochondria but instead hitch a ride with late endosomes towards the soma, where they are then lysosomally degraded (Fig. 2C lower part). The absence of Syntaphilin at mitochondria should then allow for damaged mitochondria to be moved out of the axons. Testing their observation in PD and ALS mouse models, the authors were able to observe the same decrease in Syntaphilin levels [187].

## Mitostasis in neurons

Taken together, the previous chapters highlight the neuron-specific adaptions mitochondrial biogenesis and degradation have to undergo to support the extended morphology in neurons. While mitostasis is essential for all cell types, neurons are especially dependent on healthy mitochondria. Neurons are post-mitotic and, once fully developed, are limited in their numbers as neurogenesis in adults takes place scarcely [188, 189], facing them with the challenge to either function properly or be subjected to cell death. Reduced mitochondrial quality control, ranging from mitochondrial proteases to MDVs and mitophagy, will impact neurons more than most other cell types. Similarly, failure to transport mitochondria, and the mRNAs associated with them, will over time lead to the accumulation of dysfunctional organelles in the periphery [26]. Some mitochondrial damage may be repaired locally, e.g. through local translation of mitochondrial components, including PINK1 with its multifaceted downstream targets that further enhance local translation [79], activate the UPR_mt_ via phosphorylation of HTRA2 [136], and by initiating the removal of dysfunctional proteins via MDVs [186]. Only in the most extreme cases, PINK1 may actually activate mitophagy, as under basal conditions the amount of PINK1-dependent mitophagy in neurons is negligible [149]. This is all sustained by the transport of its mRNA via mitochondrial mRNA hitch-hiking [60] and its local translation, again showing the importance of local translation for mitostasis in neurons. Several questions remain to be solved, for example how communication with the nucleus is orchestrated in neurons. Retrograde transport of locally-synthesized transcription factors such as ATF4 upon activation of the UPR_mt_/ISR can mediate this communication, but mitochondria also have been reported to form direct contact sites with the nucleus that sustain pro-survival signaling [190]. Do retrogradely travelling mitochondria serve as sentinels that bring news from the periphery? And is a directed transport of replacement mitochondria into affected areas possible or will all transcriptional measures remain a global response? Only future research will be able to answer these questions.

## Data Availability

Not applicable.

## References

[1] Gray MW (2012) Mitochondrial evolution. Cold Spring Harb Perspect Biol. 10.1101/cshperspect.a011403 22952398 10.1101/cshperspect.a011403 PMC3428767

[2] Miller KE, Sheetz MP (2004) Axonal mitochondrial transport and potential are correlated. J Cell Sci 117:2791–2804. 10.1242/jcs.01130 15150321 10.1242/jcs.01130

[3] Rangaraju V, Lauterbach M, Schuman EM (2019) Spatially stable mitochondrial compartments fuel local translation during plasticity. Cell 176:73-84.e15. 10.1016/j.cell.2018.12.013 30612742 10.1016/j.cell.2018.12.013

[4] Palmer CS, Osellame LD, Stojanovski D, Ryan MT (2011) The regulation of mitochondrial morphology: intricate mechanisms and dynamic machinery. Cell Signal 23:1534–1545. 10.1016/j.cellsig.2011.05.021 21683788 10.1016/j.cellsig.2011.05.021

[5] Otera H, Wang C, Cleland MM, Setoguchi K, Yokota S, Youle RJ, Mihara K (2010) Mff is an essential factor for mitochondrial recruitment of Drp1 during mitochondrial fission in mammalian cells. J Cell Biol 191:1141–1158. 10.1083/jcb.201007152 21149567 10.1083/jcb.201007152 PMC3002033

[6] Cipolat S, Martins De Brito O, Zilio BD, Scorrano L (2004) OPA1 requires mitofusin 1 to promote mitochondrial fusion. Proc Natl Acad Sci U S A 101:15927–15932. 10.1073/pnas.0407043101 15509649 10.1073/pnas.0407043101 PMC528769

[7] Chen H, Detmer SA, Ewald AJ, Griffin EE, Fraser SE, Chan DC (2003) Mitofusins Mfn1 and Mfn2 coordinately regulate mitochondrial fusion and are essential for embryonic development. J Cell Biol 160:189–200. 10.1083/jcb.200211046 12527753 10.1083/jcb.200211046 PMC2172648

[8] Lewis TL, Kwon SK, Lee A, Shaw R, Polleux F (2018) Mff-dependent mitochondrial fission regulates presynaptic release and axon branching by limiting axonal mitochondria size. Nat Commun. 10.1038/s41467-018-07416-2 30479337 10.1038/s41467-018-07416-2 PMC6258764

[9] Matsumoto N, Hori I, Kajita MK, Murase T, Nakamura W, Tsuji T, Miyake S, Inatani M, Konishi Y (2022) Intermitochondrial signaling regulates the uniform distribution of stationary mitochondria in axons. Mol Cell Neurosci. 10.1016/j.mcn.2022.103704 35131465 10.1016/j.mcn.2022.103704

[10] Obashi K, Okabe S (2013) Regulation of mitochondrial dynamics and distribution by synapse position and neuronal activity in the axon. Eur J Neurosci 38:2350–2363. 10.1111/ejn.12263 23725294 10.1111/ejn.12263

[11] Ohno N, Kidd GJ, Mahad D, Kiryu-Seo S, Avishai A, Komuro H, Trapp BD (2011) Myelination and axonal electrical activity modulate the distribution and motility of mitochondria at CNS nodes of Ranvier. J Neurosci 31:7249–7258. 10.1523/JNEUROSCI.0095-11.2011 21593309 10.1523/JNEUROSCI.0095-11.2011 PMC3139464

[12] Smit-Rigter L, Rajendran R, Silva CAP, Spierenburg L, Groeneweg F, Ruimschotel EM, van Versendaal D, van der Togt C, Eysel UT, Heimel JA, Lohmann C, Levelt CN (2016) Mitochondrial dynamics in visual cortex are limited in vivo and not affected by axonal structural plasticity. Curr Biol 26:2609–2616. 10.1016/j.cub.2016.07.033 27641766 10.1016/j.cub.2016.07.033

[13] Zheng X, Boyer L, Jin M, Mertens J, Kim Y, Ma L, Hamm M, Gage FH, Hunter T (2016) Metabolic reprogramming during neuronal differentiation from aerobic glycolysis to neuronal oxidative phosphorylation. Elife 5:e13374. 10.7554/eLife.13374.001 27282387 10.7554/eLife.13374 PMC4963198

[14] Pathak D, Shields LY, Mendelsohn BA, Haddad D, Lin W, Gerencser AA, Kim H, Brand MD, Edwards RH, Nakamura K (2015) The role of mitochondrially derived ATP in synaptic vesicle recycling. J Biol Chem 290:22325–22336. 10.1074/jbc.M115.656405 26126824 10.1074/jbc.M115.656405 PMC4566209

[15] Ashrafi G, Wu Z, Farrell RJ, Ryan TA (2017) GLUT4 mobilization supports energetic demands of active synapses. Neuron 93(3):606-615.e3. 10.1016/j.neuron.2016.12.020 28111082 10.1016/j.neuron.2016.12.020 PMC5330257

[16] Billups B, Forsythe ID (2002) Presynaptic mitochondrial calcium sequestration influences transmission at mammalian central synapses. J Neurosci 22:5840–5847. 10.1523/JNEUROSCI.22-14-05840.2002 12122046 10.1523/JNEUROSCI.22-14-05840.2002 PMC6757942

[17] Kwon SK, Sando R, Lewis TL, Hirabayashi Y, Maximov A, Polleux F (2016) LKB1 regulates mitochondria-dependent presynaptic calcium clearance and neurotransmitter release properties at excitatory synapses along cortical axons. PLoS Biol 14:1–27. 10.1371/journal.pbio.1002516 10.1371/journal.pbio.1002516 PMC4948842 27429220

[18] Courchet J, Lewis TL, Lee S, Courchet V, Liou DY, Aizawa S, Polleux F (2013) Terminal axon branching is regulated by the LKB1-NUAK1 kinase pathway via presynaptic mitochondrial capture. Cell 153:1510. 10.1016/j.cell.2013.05.021 23791179 10.1016/j.cell.2013.05.021 PMC3729210

[19] Spillane M, Ketschek A, Merianda TT, Twiss JL, Gallo G (2013) Mitochondria coordinate sites of axon branching through localized intra-axonal protein synthesis. Cell Rep 5:1564–1575. 10.1016/j.celrep.2013.11.022 24332852 10.1016/j.celrep.2013.11.022 PMC3947524

[20] Gutnick A, Banghart MR, West ER, Schwarz TL (2019) The light-sensitive dimerizer zapalog reveals distinct modes of immobilization for axonal mitochondria. Nat Cell Biol 21:768–777. 10.1038/s41556-019-0317-2 31061466 10.1038/s41556-019-0317-2 PMC6662610

[21] Pekkurnaz G, Wang X (2022) Mitochondrial heterogeneity and homeostasis through the lens of a neuron. Nat Metab 4:802–812. 10.1038/s42255-022-00594-w 35817853 10.1038/s42255-022-00594-w PMC11151822

[22] Stowers RS, Megeath LJ, Gó Rska-Andrzejak J, Meinertzhagen IA, Schwarz TL (2002) Axonal transport of mitochondria to synapses depends on Milton, a novel Drosophila protein. Neuron 36:1063–1077. 10.1016/s0896-6273(02)01094-2 12495622 10.1016/s0896-6273(02)01094-2

[23] Brickley K, Stephenson FA (2011) Trafficking kinesin protein (TRAK)-mediated transport of mitochondria in axons of hippocampal neurons. J Biol Chem 286:18079–18092. 10.1074/jbc.M111.236018 21454691 10.1074/jbc.M111.236018 PMC3093881

[24] Glater EE, Megeath LJ, Stowers RS, Schwarz TL (2006) Axonal transport of mitochondria requires milton to recruit kinesin heavy chain and is light chain independent. J Cell Biol 173:545–557. 10.1083/jcb.200601067 16717129 10.1083/jcb.200601067 PMC2063864

[25] Ohno N, Chiang H, Mahad DJ, Kidd GJ, Liu LP, Ransohoff RM, Sheng ZH, Komuro H, Trapp BD (2014) Mitochondrial immobilization mediated by syntaphilin facilitates survival of demyelinated axons. Proc Natl Acad Sci U S A 111:9953–9958. 10.1073/pnas.1401155111 24958879 10.1073/pnas.1401155111 PMC4103317

[26] Misgeld T, Schwarz TL (2017) Mitostasis in neurons: maintaining mitochondria in an extended cellular architecture. Neuron 96:651–666. 10.1016/j.neuron.2017.09.055 29096078 10.1016/j.neuron.2017.09.055 PMC5687842

[27] Rangaraju V, Dieck SS, Schuman EM (2017) Local translation in neuronal compartments: how local is local? EMBO Rep 18:693–711. 10.15252/embr.201744045 28404606 10.15252/embr.201744045 PMC5412868

[28] Ferree AW, Trudeau K, Zik E, Benador IY, Twig G, Gottlieb RA, Shirihai OS (2013) Mitotimer probe reveals the impact of autophagy, fusion, and motility on subcellular distribution of young and old mitochondrial protein and on relative mitochondrial protein age. Autophagy 9:1887–1896. 10.4161/auto.26503 24149000 10.4161/auto.26503 PMC4028338

[29] Hall CN, Klein-Flügge MC, Howarth C, Attwell D (2012) Oxidative phosphorylation, not glycolysis, powers presynaptic and postsynaptic mechanisms underlying brain information processing. J Neurosci 32:8940–8951. 10.1523/JNEUROSCI.0026-12.2012 22745494 10.1523/JNEUROSCI.0026-12.2012 PMC3390246

[30] Agostini M, Romeo F, Inoue S, Niklison-Chirou MV, Elia AJ, Dinsdale D, Morone N, Knight RA, Mak TW, Melino G (2016) Metabolic reprogramming during neuronal differentiation. Cell Death Differ 23:1502–1514. 10.1038/cdd.2016.36 27058317 10.1038/cdd.2016.36 PMC5072427

[31] Iwata R, Casimir P, Vanderhaeghen P (2020) Mitochondrial dynamics in postmitotic cells regulate neurogenesis. Science 369:858–862. 10.1126/science.aba9760 32792401 10.1126/science.aba9760

[32] Ishihara N, Nomura M, Jofuku A, Kato H, Suzuki SO, Masuda K, Otera H, Nakanishi Y, Nonaka I, Goto YI, Taguchi N, Morinaga H, Maeda M, Takayanagi R, Yokota S, Mihara K (2009) Mitochondrial fission factor Drp1 is essential for embryonic development and synapse formation in mice. Nat Cell Biol 11:958–966. 10.1038/ncb1907 19578372 10.1038/ncb1907

[33] Bandeira F, Lent R, Herculano-Houzel S (2009) Changing numbers of neuronal and non-neuronal cells underlie postnatal brain growth in the rat. Proc Natl Acad Sci U S A 106:14108–14113. 10.1073/pnas.0804650106 19666520 10.1073/pnas.0804650106 PMC2729028

[34] Chen H, McCaffery JM, Chan DC (2007) Mitochondrial fusion protects against neurodegeneration in the cerebellum. Cell 130:548–562. 10.1016/j.cell.2007.06.026 17693261 10.1016/j.cell.2007.06.026

[35] Ando M, Hashiguchi A, Okamoto Y, Yoshimura A, Hiramatsu Y, Yuan J, Higuchi Y, Mitsui J, Ishiura H, Umemura A, Maruyama K, Matsushige T, Morishita S, Nakagawa M, Tsuji S, Takashima H (2017) Clinical and genetic diversities of Charcot-Marie-Tooth disease with MFN2 mutations in a large case study. J Peripher Nerv Syst 22:191–199. 10.1111/jns.12228 28660751 10.1111/jns.12228 PMC5697682

[36] Kijima K, Numakura C, Izumino H, Umetsu K, Nezu A, Shiiki T, Ogawa M, Ishizaki Y, Kitamura T, Shozawa Y, Hayasaka K (2005) Mitochondrial GTPase mitofusin 2 mutation in Charcot-Marie-Tooth neuropathy type 2A. Hum Genet 116:23–27. 10.1007/s00439-004-1199-2 15549395 10.1007/s00439-004-1199-2

[37] Misko A, Jiang S, Wegorzewska I, Milbrandt J, Baloh RH (2010) Mitofusin 2 is necessary for transport of axonal mitochondria and interacts with the Miro/Milton complex. J Neurosci 30:4232–4240. 10.1523/JNEUROSCI.6248-09.2010 20335458 10.1523/JNEUROSCI.6248-09.2010 PMC2852190

[38] Rath S, Sharma R, Gupta R, Ast T, Chan C, Durham TJ, Goodman RP, Grabarek Z, Haas ME, Hung WHW, Joshi PR, Jourdain AA, Kim SH, Kotrys AV, Lam SS, McCoy JG, Meisel JD, Miranda M, Panda A, Patgiri A, Rogers R, Sadre S, Shah H, Skinner OS, To TL, Walker MA, Wang H, Ward PS, Wengrod J, Yuan CC, Calvo SE, Mootha VK (2021) MitoCarta3.0: an updated mitochondrial proteome now with sub-organelle localization and pathway annotations. Nucleic Acids Res 49(D1):D1541–D1547. 10.1093/nar/gkaa1011 33174596 10.1093/nar/gkaa1011 PMC7778944

[39] Alston CL, Rocha MC, Lax NZ, Turnbull DM, Taylor RW (2017) The genetics and pathology of mitochondrial disease. J Pathol 241:236–250. 10.1002/path.4809 27659608 10.1002/path.4809 PMC5215404

[40] Baker ZN, Forny P, Pagliarini DJ (2024) Mitochondrial proteome research: the road ahead. Nat Rev Mol Cell Biol 25:65–82. 10.1038/s41580-023-00650-7 37773518 10.1038/s41580-023-00650-7 PMC11378943

[41] Harbauer AB, Zahedi RP, Sickmann A, Pfanner N, Meisinger C (2014) The protein import machinery of mitochondria - a regulatory hub in metabolism, stress, and disease. Cell Metab 19:357–372. 10.1016/j.cmet.2014.01.010 24561263 10.1016/j.cmet.2014.01.010

[42] Brix J, Dietmeier K, Pfanner N (1997) Differential recognition of preproteins by the purified cytosolic domains of the mitochondrial import receptors Tom20, Tom22, and Tom70*. J Biol Chem 272:20730–20735. 10.1074/jbc.272.33.20730 9252394 10.1074/jbc.272.33.20730

[43] Mihara K, Omura T (1996) Cytoplasmic chaperones in precursor targeting to mitochondria: the role of MSF and hsp70. Trends Cell Biol 6:104–108. 10.1016/0962-8924(96)81000-2 15157486 10.1016/0962-8924(96)81000-2

[44] Stuart RA, Cyr DM, Craig EA, Neupert W (1994) Mitochondrial molecular chaperones: their role in protein translocation. Trends Biochem Sci 19:87–92. 10.1016/0968-0004(94)90041-8 8160272 10.1016/0968-0004(94)90041-8

[45] Arceo XG, Koslover EF, Zid BM, Brown AI (2022) Mitochondrial mRNA localization is governed by translation kinetics and spatial transport. PLoS Comput Biol. 10.1371/journal.pcbi.1010413 35984860 10.1371/journal.pcbi.1010413 PMC9432724

[46] Segura I, Harbauer A (2024) The role of mitochondrial RNA association for mitochondrial homeostasis in neurons. Biochem J 481:119–139. 10.1042/BCJ20230110

[47] Hémono M, Haller A, Chicher J, Duchêne AM, Ngondo RP (2022) The interactome of CLUH reveals its association to SPAG5 and its co-translational proximity to mitochondrial proteins. BMC Biol 20:13. 10.1186/s12915-021-01213-y 35012549 10.1186/s12915-021-01213-y PMC8744257

[48] Eliyahu E, Pnueli L, Melamed D, Scherrer T, Gerber AP, Pines O, Rapaport D, Arava Y (2010) Tom20 mediates localization of mRNAs to mitochondria in a translation-dependent manner. Mol Cell Biol 30:284–294. 10.1128/mcb.00651-09 19858288 10.1128/MCB.00651-09 PMC2798288

[49] El Zawily AM, Schwarzländer M, Finkemeier I, Johnston IG, Benamar A, Cao Y, Gissot C, Meyer AJ, Wilson K, Datla R, Macherel D, Jones NS, Logan DC (2014) Friendly regulates mitochondrial distribution, fusion, and quality control in Arabidopsis. Plant Physiol 166:808–828. 10.1104/pp.114.243824 25165398 10.1104/pp.114.243824 PMC4213110

[50] Bykov YS, Rapaport D, Herrmann JM, Schuldiner M (2020) Cytosolic events in the biogenesis of mitochondrial proteins. Trends Biochem Sci 45:650–667. 10.1016/j.tibs.2020.04.001 32409196 10.1016/j.tibs.2020.04.001

[51] Zhu Z, Mallik S, Stevens TA, Huang R, Levy ED, Shan S (2025) Principles of cotranslational mitochondrial protein import. Cell 188:5605-5617.e14. 10.1016/j.cell.2025.07.021 40795856 10.1016/j.cell.2025.07.021 PMC12396113

[52] Luo J, Khandwala S, Hu J, Lee SY, Hickey KL, Levine ZG, Harper JW, Ting AY, Weissman JS (2025) Proximity-specific ribosome profiling reveals the logic of localized mitochondrial translation. Cell 188:5589-5604.e17. 10.1016/j.cell.2025.08.002 40876456 10.1016/j.cell.2025.08.002 PMC12650760

[53] Gabrovsek L, Collins KB, Aggarwal S, Saunders LM, Lau HT, Suh D, Sancak Y, Trapnell C, Ong SE, Smith FD, Scott JD (2020) A-kinase-anchoring protein 1 (dAKAP1)-based signaling complexes coordinate local protein synthesis at the mitochondrial surface. J Biol Chem 295:10749–10765. 10.1074/jbc.ra120.013454 32482893 10.1074/jbc.RA120.013454 PMC7397098

[54] Zilio E, Schlegel T, Zaninello M, Rugarli E (2025) The role of mitochondrial mRNA translation in cellular communication. J Cell Sci. 10.1242/jcs.263753 40326563 10.1242/jcs.263753

[55] Zaninello M, Schlegel T, Nolte H, Pirzada M, Savino E, Barth E, Klein I, Wüstenberg H, Uddin T, Wolff L, Wirth B, Lehmann HC, Cioni J-M, Langer T, Rugarli EI (2024) Cluh maintains functional mitochondria and translation in motoneuronal axons and prevents peripheral neuropathy. Sci Adv. 10.1126/sciadv.adn2050 38809982 10.1126/sciadv.adn2050 PMC11135423

[56] Gao J, Schatton D, Martinelli P, Hansen H, Pla-Martin D, Barth E, Becker C, Altmueller J, Frommolt P, Sardiello M, Rugarli EI (2014) Cluh regulates mitochondrial biogenesis by binding mRNAs of nuclear-encoded mitochondrial proteins. J Cell Biol 207:213–223. 10.1083/jcb.201403129 25349259 10.1083/jcb.201403129 PMC4210445

[57] Schatton D, Pla-Martin D, Marx MC, Hansen H, Mourier A, Nemazanyy I, Pessia A, Zentis P, Corona T, Kondylis V, Barth E, Schauss AC, Velagapudi V, Rugarli EI (2017) Cluh regulates mitochondrial metabolism by controlling translation and decay of target mRNAs. J Cell Biol 216:675–693. 10.1083/jcb.201607019 28188211 10.1083/jcb.201607019 PMC5350512

[58] Sen A, Rodriguez-Martinez A, Young-Baird SK, Cox RT (2024) The *Drosophila* ribonucleoprotein Clueless is required for ribosome biogenesis in vivo. J Biol Chem. 10.1016/j.jbc.2024.107946 39481601 10.1016/j.jbc.2024.107946 PMC11625335

[59] Loedige I, Baranovskii A, Mendonsa S, Dantsuji S, Popitsch N, Breimann L, Zerna N, Cherepanov V, Milek M, Ameres S, Chekulaeva M (2023) mRNA stability and m6A are major determinants of subcellular mRNA localization in neurons. Mol Cell 83:2709-2725.e10. 10.1016/j.molcel.2023.06.021 37451262 10.1016/j.molcel.2023.06.021 PMC10529935

[60] Harbauer AB, Hees JT, Wanderoy S, Segura I, Gibbs W, Cheng Y, Ordonez M, Cai Z, Cartoni R, Ashrafi G, Wang C, Perocchi F, He Z, Schwarz TL (2022) Neuronal mitochondria transport Pink1 mRNA via synaptojanin 2 to support local mitophagy. Neuron 110:1–16. 10.1016/j.neuron.2022.01.035 35216662 10.1016/j.neuron.2022.01.035 PMC9081165

[61] Cohen B, Altman T, Golani-Armon A, Savulescu AF, Ibraheem A, Mhlanga MM, Perlson E, Arava YS (2022) The nuclear encoded Cox7c mRNA co-transport with mitochondria along axons via coding-region dependent mechanism. J Cell Sci 135:259436. 10.1242/jcs.259436 10.1242/jcs.259436 PMC9481926 35833493

[62] Cioni JM, Lin JQ, Holtermann AV, Koppers M, Jakobs MAH, Azizi A, Turner-Bridger B, Shigeoka T, Franze K, Harris WA, Holt CE (2019) Late endosomes act as mRNA translation platforms and sustain mitochondria in axons. Cell 176:56-72.e15. 10.1016/j.cell.2018.11.030 30612743 10.1016/j.cell.2018.11.030 PMC6333918

[63] De Pace R, Ghosh S, Ryan VH, Sohn M, Jarnik M, Rezvan Sangsari P, Morgan NY, Dale RK, Ward ME, Bonifacino JS (2024) Messenger RNA transport on lysosomal vesicles maintains axonal mitochondrial homeostasis and prevents axonal degeneration. Nat Neurosci 27:1087–1102. 10.1038/s41593-024-01619-1 38600167 10.1038/s41593-024-01619-1 PMC11156585

[64] Schuhmacher JS, tom Dieck S, Christoforidis S, Landerer C, Davila Gallesio J, Hersemann L, Seifert S, Schäfer R, Giner A, Toth-Petroczy A, Kalaidzidis Y, Bohnsack KE, Bohnsack MT, Schuman EM, Zerial M (2023) The Rab5 effector FERRY links early endosomes with mRNA localization. Mol Cell 83:1839-1855.e13. 10.1016/j.molcel.2023.05.012 37267905 10.1016/j.molcel.2023.05.012

[65] Cosker KE, Pazyra-Murphy MF, Fenstermacher SJ, Segal RA (2013) Target-derived neurotrophins coordinate transcription and transport of Bclw to prevent axonal degeneration. Ann Intern Med 158:5195–5207. 10.1523/JNEUROSCI.3862-12.2013 10.1523/JNEUROSCI.3862-12.2013 PMC3866501 23516285

[66] Fenton AR, Peng R, Bond C, Hugelier S, Lakadamyali M, Chang YW, Holzbaur ELF, Jongens TA (2024) FMRP regulates MFF translation to locally direct mitochondrial fission in neurons. Nat Cell Biol 26:2061–2074. 10.1038/s41556-024-01544-2 39548330 10.1038/s41556-024-01544-2 PMC11628401

[67] Lin W, Kang UJ (2008) Characterization of PINK1 processing, stability, and subcellular localization. J Neurochem 106:464–474. 10.1111/j.1471-4159.2008.05398.x 18397367 10.1111/j.1471-4159.2008.05398.x PMC3638740

[68] Lazarou M, Jin SM, Kane LA, Youle RJ (2012) Role of PINK1 binding to the TOM complex and alternate intracellular membranes in recruitment and activation of the E3 ligase Parkin. Dev Cell 22:320–333. 10.1016/j.devcel.2011.12.014 22280891 10.1016/j.devcel.2011.12.014 PMC3288275

[69] Ando M, Fiesel FC, Hudec R, Caulfield TR, Ogaki K, Górka-Skoczylas P, Koziorowski D, Friedman A, Chen L, Dawson VL, Dawson TM, Bu G, Ross OA, Wszolek ZK, Springer W (2017) The PINK1 p. I368N mutation affects protein stability and ubiquitin kinase activity. Mol Neurodegener. 10.1186/s13024-017-0174-z 28438176 10.1186/s13024-017-0174-z PMC5404317

[70] Nemoto Y, De Camilli P (1999) Recruitment of an alternatively spliced form of synaptojanin 2 to mitochondria by the interaction with the PDZ domain of a mitochondrial outer membrane protein. EMBO J 18:2991–3006. 10.1093/emboj/18.11.2991 10357812 10.1093/emboj/18.11.2991 PMC1171381

[71] Nemoto Y, Wenk MR, Watanabe M, Daniell L, Murakami T, Ringstad N, Yamada H, Takei K, De Camilli P (2001) Identification and characterization of a Synaptojanin 2 splice isoform predominantly expressed in nerve terminals. J Biol Chem 276:41133–41142. 10.1074/jbc.M106404200 11498538 10.1074/jbc.M106404200

[72] Biever A, Glock C, Tushev G, Ciirdaeva E, Dalmay T, Langer JD, Schuman EM (2020) Monosomes actively translate synaptic mRNAs in neuronal processes. Science. 10.1126/science.aay4991 32001627 10.1126/science.aay4991

[73] Koppers M, Özkan N, Nguyen HH, Jurriens D, McCaughey J, Nguyen DTM, Li CH, Stucchi R, Altelaar M, MacGillavry HD, Kapitein LC, Hoogenraad CC, Farías GG (2024) Axonal endoplasmic reticulum tubules control local translation via P180/RRBP1-mediated ribosome interactions. Dev Cell 59:2053-2068.e9. 10.1016/j.devcel.2024.05.005 38815583 10.1016/j.devcel.2024.05.005 PMC11338522

[74] Reid DW, Nicchitta CV (2015) Diversity and selectivity in mRNA translation on the endoplasmic reticulum. Nat Rev Mol Cell Biol 16:221–231. 10.1038/nrm3958 25735911 10.1038/nrm3958 PMC4494666

[75] Hees JT, Segura I, Schneider A, Schifferer M, Misgeld T, Harbauer AB (2024) ER-associated biogenesis of PINK1 preprotein for neuronal mitophagy. bioRxiv preprint. 10.1101/2024.06.21.600039

[76] Hees JT, Wanderoy S, Lindner J, Helms M, Murali Mahadevan H, Harbauer AB (2024) Insulin signalling regulates Pink1 mRNA localization via modulation of AMPK activity to support PINK1 function in neurons. Nat Metab 6:514–530. 10.1038/s42255-024-01007-w 38504131 10.1038/s42255-024-01007-w PMC10963278

[77] Hansen KG, Aviram N, Laborenz J, Bibi C, Meyer M, Spang A, Schuldiner M, Herrmann JM (2018) An ER surface retrieval pathway safeguards the import of mitochondrial membrane proteins in yeast. Science 361:1118–1122. 10.1126/science.aar8174 30213914 10.1126/science.aar8174

[78] Choi H, Liao Y-C, Yoon YJ, Grimm J, Lavis LD, Singer RH, Lippincott-Schwartz J (2023) Lysosomal release of amino acids at ER three-way junctions regulates transmembrane and secretory protein mRNA translation. bioRxiv preprint. 10.1101/2023.08.01.551382

[79] Gehrke S, Wu Z, Klinkenberg M, Sun Y, Auburger G, Guo S, Lu B (2015) PINK1 and parkin control localized translation of respiratory chain component mRNAs on mitochondria outer membrane. Cell Metab 21:95–108. 10.1016/j.cmet.2014.12.007 25565208 10.1016/j.cmet.2014.12.007 PMC4455944

[80] Zhang Y, Wang ZH, Liu Y, Chen Y, Sun N, Gucek M, Zhang F, Xu H (2019) PINK1 inhibits local protein synthesis to limit transmission of deleterious mitochondrial DNA mutations. Mol Cell 73:1127-1137.e5. 10.1016/j.molcel.2019.01.013 30772175 10.1016/j.molcel.2019.01.013 PMC7485087

[81] Zhang Y, Chen Y, Gucek M, Xu H (2016) The mitochondrial outer membrane protein MDI promotes local protein synthesis and mt DNA replication. EMBO J 35:1045–1057. 10.15252/embj.201592994 27053724 10.15252/embj.201592994 PMC4868955

[82] Lewis BM, Cho CY, Her H-L, Mizrahi O, Hunter T, Yeo GW (2024) Larp4 is an RNA-binding protein that binds nuclear-encoded mitochondrial mrnas to promote mitochondrial function. RNA 30:223–239. 10.1261/rna.079799.123 38164626 10.1261/rna.079799.123 PMC10870378

[83] Huang E, Qu D, Huang T, Rizzi N, Boonying W, Krolak D, Ciana P, Woulfe J, Klein C, Slack RS, Figeys D, Park DS (2017) PINK1-mediated phosphorylation of LETM1 regulates mitochondrial calcium transport and protects neurons against mitochondrial stress. Nat Commun. 10.1038/s41467-017-01435-1 29123128 10.1038/s41467-017-01435-1 PMC5680261

[84] Kostic M, Ludtmann MHR, Bading H, Hershfinkel M, Steer E, Chu CT, Abramov AY, Sekler I (2015) PKA phosphorylation of NCLX reverses mitochondrial calcium overload and depolarization, promoting survival of PINK1-deficient dopaminergic neurons. Cell Rep 13:376–386. 10.1016/j.celrep.2015.08.079 26440884 10.1016/j.celrep.2015.08.079 PMC4709126

[85] Gandhi S, Wood-Kaczmar A, Yao Z, Plun-Favreau H, Deas E, Klupsch K, Downward J, Latchman DS, Tabrizi SJ, Wood NW, Duchen MR, Abramov AY (2009) PINK1-associated Parkinson’s disease is caused by neuronal vulnerability to calcium-induced cell death. Mol Cell 33:627–638. 10.1016/j.molcel.2009.02.013 19285945 10.1016/j.molcel.2009.02.013 PMC2724101

[86] Kleele T, Rey T, Winter J, Zaganelli S, Mahecic D, Perreten Lambert H, Ruberto FP, Nemir M, Wai T, Pedrazzini T, Manley S (2021) Distinct fission signatures predict mitochondrial degradation or biogenesis. Nature 593:435–439. 10.1038/s41586-021-03510-6 33953403 10.1038/s41586-021-03510-6

[87] Wong YC, Ysselstein D, Krainc D (2018) Mitochondria-lysosome contacts regulate mitochondrial fission via RAB7 GTP hydrolysis. Nature 554:382–386. 10.1038/nature25486 29364868 10.1038/nature25486 PMC6209448

[88] Kuzniewska B, Cysewski D, Wasilewski M, Sakowska P, Milek J, Kulinski TM, Winiarski M, Kozielewicz P, Knapska E, Dadlez M, Chacinska A, Dziembowski A, Dziembowska M (2020) Mitochondrial protein biogenesis in the synapse is supported by local translation. EMBO Rep. 10.15252/embr.201948882 32558077 10.15252/embr.201948882 PMC7403725

[89] Aschrafi A, Natera-Naranjo O, Gioio AE, Kaplan BB (2010) Regulation of axonal trafficking of cytochrome c oxidase IV mRNA. Mol Cell Neurosci 43:422–430. 10.1016/j.mcn.2010.01.009 20144716 10.1016/j.mcn.2010.01.009 PMC2845174

[90] Kar AN, Vargas JNS, Chen CY, Kowalak JA, Gioio AE, Kaplan BB (2017) Molecular determinants of cytochrome C oxidase IV mRNA axonal trafficking. Mol Cell Neurosci 80:32–43. 10.1016/j.mcn.2017.01.008 28161363 10.1016/j.mcn.2017.01.008 PMC5393917

[91] Hillefors M, Gioio AE, Mameza MG, Kaplan BB (2007) Axon viability and mitochondrial function are dependent on local protein synthesis in sympathetic neurons. Cell Mol Neurobiol 27:701–716. 10.1007/s10571-007-9148-y 17619140 10.1007/s10571-007-9148-y PMC11517218

[92] Harbauer AB (2017) Mitochondrial health maintenance in axons. Biochem Soc Trans 45:1045–1052. 10.1042/BST20170023 28778985 10.1042/BST20170023

[93] Bomba-Warczak E, Edassery SL, Hark TJ, Savas JN (2021) Long-lived mitochondrial cristae proteins in mouse heart and brain. J Cell Biol. 10.1083/jcb.202005193 34259807 10.1083/jcb.202005193 PMC8282663

[94] McShane E, Couvillion M, Ietswaart R, Prakash G, Smalec BM, Soto I, Baxter-Koenigs AR, Choquet K, Churchman LS (2024) A kinetic dichotomy between mitochondrial and nuclear gene expression processes. Mol Cell 84:1541-1555.e11. 10.1016/j.molcel.2024.02.028 38503286 10.1016/j.molcel.2024.02.028 PMC11236289

[95] Hirabayashi Y, Lewis TL, Du Y, Virga DM, Decker AM, Coceano G, Alvelid J, Paul MA, Hamilton S, Kneis P, Takahashi Y, Gaublomme JT, Testa I, Polleux F (2024) Most axonal mitochondria in cortical pyramidal neurons lack mitochondrial DNA and consume ATP. bioRxiv preprint. 10.1101/2024.02.12.579972

[96] Stauch KL, Purnell PR, Fox HS (2014) Quantitative proteomics of synaptic and nonsynaptic mitochondria: insights for synaptic mitochondrial vulnerability. J Proteome Res 13:2620–2636. 10.1021/pr500295n 24708184 10.1021/pr500295n PMC4015687

[97] Pastor AM, Lewis LSC, Müller S, Guedes-Dias P, Olifiers L, Pelkonen P, del Rio Martin A, Fecher C, Skiba NP, Hao Y, Gavoci A, Trovo L, Aktas MA, Mahadevan HM, Harbauer AB, Bomze HM, Arshavsky VY, Brill MS, Cartoni R, Lichtenthaler SF, Gospe SM, Misgeld T (2025) Neuronal compartmentalization results in “impoverished” axonal mitochondria. bioRxiv preprint. 10.1101/2025.10.27.684882

[98] Yousefi R, Fornasiero EF, Cyganek L, Montoya J, Jakobs S, Rizzoli SO, Rehling P, Pacheu-Grau D (2021) Monitoring mitochondrial translation in living cells. EMBO Rep. 10.15252/embr.202051635 33586863 10.15252/embr.202051635 PMC8024989

[99] Lee S, Park D, Lim C, Kim JI, Min KT (2022) MtIF3 is locally translated in axons and regulates mitochondrial translation for axonal growth. BMC Biol. 10.1186/s12915-021-01215-w 34996455 10.1186/s12915-021-01215-w PMC8742369

[100] Lewis TL, Turi GF, Kwon SK, Losonczy A, Polleux F (2016) Progressive decrease of mitochondrial motility during maturation of cortical axons in vitro and in vivo. Curr Biol 26:2602–2608. 10.1016/j.cub.2016.07.064 27641765 10.1016/j.cub.2016.07.064 PMC5235338

[101] Takihara Y, Inatani M, Eto K, Inoue T, Kreymerman A, Miyake S, Ueno S, Nagaya M, Nakanishi A, Iwao K, Takamura Y, Sakamoto H, Satoh K, Kondo M, Sakamoto T, Goldberg JL, Nabekura J, Tanihara H (2015) In vivo imaging of axonal transport of mitochondria in the diseased and aged mammalian CNS. Proc Natl Acad Sci U S A 112:10515–10520. 10.1073/pnas.1509879112 26240337 10.1073/pnas.1509879112 PMC4547257

[102] Cardanho-Ramos C, Simões RA, Wang YZ, Faria-Pereira A, Bomba-Warczak E, Craessaerts K, Spinazzi M, Savas JN, Morais VA (2024) Local mitochondrial replication in the periphery of neurons requires the eEF1A1 protein and thetranslation of nuclear-encoded proteins. iScience 27:109136. 10.1016/j.isci.2024.109136 38510136 10.1016/j.isci.2024.109136 PMC10951640

[103] Tatsuta T, Langer T (2008) Quality control of mitochondria: protection against neurodegeneration and ageing. EMBO J 27:306–314. 10.1038/sj.emboj.7601972 18216873 10.1038/sj.emboj.7601972 PMC2234350

[104] Rugarli EI, Langer T (2012) Mitochondrial quality control: a matter of life and death for neurons. EMBO J 31:1336–1349. 10.1038/emboj.2012.38 22354038 10.1038/emboj.2012.38 PMC3321185

[105] Lemasters JJ (2005) Selective mitochondrial autophagy, or mitophagy, as a targeted defense against oxidative stress, mitochondrial dysfunction, and aging. Rejuvenation Res 8:3–5. 10.1089/rej.2005.8.3 15798367 10.1089/rej.2005.8.3

[106] Sugiura A, McLelland G, Fon EA, McBride HM (2014) A new pathway for mitochondrial quality control: mitochondrial-derived vesicles. EMBO J 33:2142–2156. 10.15252/embj.201488104 25107473 10.15252/embj.201488104 PMC4282503

[107] Zhao RZ, Jiang S, Zhang L, Bin Yu Z (2019) Mitochondrial electron transport chain, ROS generation and uncoupling (Review). Int J Mol Med 44:3–15. 10.3892/ijmm.2019.4188 31115493 10.3892/ijmm.2019.4188 PMC6559295

[108] Starke-Reed PE, Oliver CN (1989) Protein oxidation and proteolysis during aging and oxidative stress. Arch Biochem Biophys 275:559–567. 10.1016/0003-9861(89)90402-5 2574564 10.1016/0003-9861(89)90402-5

[109] Deshwal S, Fiedler KU, Langer T (2020) Mitochondrial proteases: multifaceted regulators of mitochondrial plasticity. Annu Rev Biochem 89:501–528. 10.1146/annurev-biochem-062917 32075415 10.1146/annurev-biochem-062917-012739

[110] Wang N, Gottesmant S, Willingham MC, Gottesman MM, Maurizi MR (1993) A human mitochondrial ATP-dependent protease that is highly homologous to bacterial Lon protease. Proc Natl Acad Sci USA 90:11247–11251. 10.1073/pnas.90.23.11247 8248235 10.1073/pnas.90.23.11247 PMC47959

[111] Katayama-Fujimura Y, Gottesman S, Maurizi MR (1987) A multiple-component, ATP-dependent protease from *Escherichia coli*. J Biol Chem 262:4477–4485. 10.1016/s0021-9258(18)61217-7 3549708

[112] Arlt H, Steglich G, Perryman R, Guiard B, Neupert W, Langer T, Arlt H, Steglich G (1998) The formation of respiratory chain complexes in mitochondria is under the proteolytic control of the m-AAA protease. EMBO J 17:4837–4847. 10.1093/emboj/17.16.4837 9707443 10.1093/emboj/17.16.4837 PMC1170813

[113] Leonhard K, Herrmann JM, Stuart RA, Mannhaupt G, Neupert W, Langer T (1996) AAA proteases with catalytic sites on opposite membrane surfaces comprise a proteolytic system for the ATP-dependent degradation of inner membrane proteins in mitochondria. EMBO J 15:4218–4229 8861950 PMC452147

[114] Weber ER, Hanekamp T, Thorsness PE (1996) Biochemical and functional analysis of the YME1 gene product, an ATP and zinc-dependent mitochondrial protease from *S. cerevisiae*. Mol Biol Cell 7:307–317. 10.1091/mbc.7.2.307 8688560 10.1091/mbc.7.2.307 PMC275881

[115] Hegde R, Srinivasula SM, Zhang Z, Wassell R, Mukattash R, Cilenti L, Dubois G, Lazebnik Y, Zervos AS, Fernandes-Alnemri T, Alnemri ES (2002) Identification of Omi/HtrA2 as a mitochondrial apoptotic serine protease that disrupts inhibitor of apoptosis protein-caspase interaction. J Biol Chem 277:432–438. 10.1074/jbc.M109721200 11606597 10.1074/jbc.M109721200

[116] He YC, Huang P, Li QQ, Sun Q, Li DH, Wang T, Shen JY, Du JJ, Cui SS, Gao C, Fu R, Chen S (2017) Mutation analysis of HTRA2 gene in Chinese familial essential tremor and familial Parkinson’s disease. Parkinsons Dis. 10.1155/2017/3217474 28243480 10.1155/2017/3217474 PMC5294371

[117] Unal Gulsuner H, Gulsuner S, Mercan FN, Onat OE, Walsh T, Shahin H, Lee MK, Dogu O, Kansu T, Topaloglu H, Elibol B, Akbostanci C, King MC, Ozcelik T, Tekinay AB (2014) Mitochondrial serine protease HTRA2 p. G399S in a kindred with essential tremor and Parkinson disease. Proc Natl Acad Sci U S A 111:18285–18290. 10.1073/pnas.1419581111 25422467 10.1073/pnas.1419581111 PMC4280582

[118] Casari G, De Fusco M, Ciarmatori S, Zeviani M, Mora M, Fernandez P, De Michele G, Filla A, Cocozza S, Marconi R, Dürr A, Fontaine B, Ballabio A (1998) Spastic paraplegia and OXPHOS impairment caused by mutations in paraplegin, a nuclear-encoded mitochondrial metalloprotease. Cell 93:973–983. 10.1016/s0092-8674(00)81203-9 9635427 10.1016/s0092-8674(00)81203-9

[119] Ferreirinha F, Quattrini A, Pirozzi M, Valsecchi V, Dina G, Broccoli V, Auricchio A, Piemonte F, Tozzi G, Gaeta L, Casari G, Ballabio A, Rugarli EI (2004) Axonal degeneration in paraplegin-deficient mice is associated with abnormal mitochondria and impairment of axonal transport. J Clin Invest 113:231–242. 10.1172/JCI200420138 14722615 10.1172/JCI20138 PMC311437

[120] Bota DA, Davies KJA (2002) Lon protease preferentially degrades oxidized mitochondrial aconitase by an ATP-stimulated mechanism. Nat Cell Biol 4:674–680. 10.1038/ncb836 12198491 10.1038/ncb836

[121] Bota DA, Ngo JK, Davies KJA (2005) Downregulation of the human Lon protease impairs mitochondrial structure and function and causes cell death. Free Radic Biol Med 38:665–677. 10.1016/j.freeradbiomed.2004.11.017 15683722 10.1016/j.freeradbiomed.2004.11.017

[122] Korhonen JA, Gaspari M, Falkenberg M (2003) TWINKLE has 5′ → 3′ DNA helicase activity and is specifically stimulated by mitochondrial single-stranded DNA-binding protein. J Biol Chem 278:48627–48632. 10.1074/jbc.M306981200 12975372 10.1074/jbc.M306981200

[123] Liu T, Lu B, Lee I, Ondrovičová G, Kutejová E, Suzuki CK (2004) DNA and RNA binding by the mitochondrial Lon protease is regulated by nucleotide and protein substrate. J Biol Chem 279:13902–13910. 10.1074/jbc.M309642200 14739292 10.1074/jbc.M309642200

[124] Meredith GE, Rademacher DJ (2011) MPTP mouse models of Parkinson’s disease: an update. J Parkinsons Dis 1:19–33. 10.3233/JPD-2011-11023 23275799 10.3233/JPD-2011-11023 PMC3530193

[125] Bulteau AL, Mena NP, Auchère F, Lee I, Prigent A, Lobsiger CS, Camadro JM, Hirsch EC (2017) Dysfunction of mitochondrial Lon protease and identification of oxidized protein in mouse brain following exposure to MPTP: implications for Parkinson disease. Free Radic Biol Med 108:236–246. 10.1016/j.freeradbiomed.2017.03.036 28365360 10.1016/j.freeradbiomed.2017.03.036

[126] Hu D, Sun X, Liao X, Zhang X, Zarabi S, Schimmer A, Hong Y, Ford C, Luo Y, Qi X (2019) Alpha-synuclein suppresses mitochondrial protease ClpP to trigger mitochondrial oxidative damage and neurotoxicity. Acta Neuropathol 137:939–960. 10.1007/s00401-019-01993-2 30877431 10.1007/s00401-019-01993-2 PMC6531426

[127] Baker TA, Sauer RT (2012) ClpXP, an ATP-powered unfolding and protein-degradation machine. Biochim Biophys Acta Mol Cell Res 1823:15–28. 10.1016/j.bbamcr.2011.06.007 10.1016/j.bbamcr.2011.06.007 PMC3209554 21736903

[128] Xie HR, Sen HuL, Li GY (2010) SH-SY5Y human neuroblastoma cell line: In vitro cell model of dopaminergic neurons in Parkinson’s disease. Chin Med J (Engl) 123:1086–1092. 10.3760/cma.j.issn.0366-6999.2010.08.021 20497720

[129] Arlt H, Tauer R, Feldmann H (1996) The YTA10-12 complex, an AAA protease with chaperone-like activity in the inner membrane of mitochondria. Cell 85:875–885. 10.1016/S0092-8674(00)81271-4 8681382 10.1016/s0092-8674(00)81271-4

[130] Banfi S, Bassi MT, Andolfi G, Marchitiello A, Zanotta S, Ballabio A, Casari G, Franco B (1999) Identification and characterization of AFG3L2, a novel paraplegin-related gene. Genomics 59:51–58. 10.1006/geno.1999.5818 10395799 10.1006/geno.1999.5818

[131] Koppen M, Metodiev MD, Casari G, Rugarli EI, Langer T (2007) Variable and tissue-specific subunit composition of mitochondrial m-AAA protease complexes linked to hereditary spastic paraplegia. Mol Cell Biol 27:758–767. 10.1128/mcb.01470-06 17101804 10.1128/MCB.01470-06 PMC1800790

[132] Nolden M, Ehses S, Koppen M, Bernacchia A, Rugarli EI, Langer T (2005) The m-AAA protease defective in hereditary spastic paraplegia controls ribosome assembly in mitochondria. Cell 123:277–289. 10.1016/j.cell.2005.08.003 16239145 10.1016/j.cell.2005.08.003

[133] Suzuki Y, Imai Y, Nakayama H, Takahashi K, Takio K, Takahashi R (2001) A serine protease, Htr A2, is released from the mitochondria and interacts with XIAP, inducing cell death. Mol Cell 8:613–621. 10.1016/s1097-2765(01)00341-0 11583623 10.1016/s1097-2765(01)00341-0

[134] Martins LM, Morrison A, Klupsch K, Fedele V, Moisoi N, Teismann P, Abuin A, Grau E, Geppert M, Livi GP, Creasy CL, Martin A, Hargreaves I, Heales SJ, Okada H, Brandner S, Schulz JB, Mak T, Downward J (2004) Neuroprotective role of the Reaper-related serine protease HtrA2/Omi revealed by targeted deletion in mice. Mol Cell Biol 24:9848–9862. 10.1128/mcb.24.22.9848-9862.2004 15509788 10.1128/MCB.24.22.9848-9862.2004 PMC525490

[135] Seong YM, Choi JY, Park HJ, Kim KJ, Ahn SG, Seong GH, Kim IK, Kang S, Rhim H (2004) Autocatalytic processing of HtrA2/Omi is essential for induction of caspase-dependent cell death through antagonizing XIAP. J Biol Chem 279:37588–37596. 10.1074/jbc.M401408200 15201285 10.1074/jbc.M401408200

[136] Plun-Favreau H, Klupsch K, Moisoi N, Gandhi S, Kjaer S, Frith D, Harvey K, Deas E, Harvey RJ, McDonald N, Wood NW, Martins ML, Downward J (2007) The mitochondrial protease HtrA2 is regulated by Parkinson’s disease-associated kinase PINK1. Nat Cell Biol 9:1243–1252. 10.1038/ncb1644 17906618 10.1038/ncb1644

[137] Guo X, Aviles G, Liu Y, Tian R, Unger BA, Lin YHT, Wiita AP, Xu K, Correia MA, Kampmann M (2020) Mitochondrial stress is relayed to the cytosol by an OMA1–DELE1–HRI pathway. Nature 579:427–432. 10.1038/s41586-020-2078-2 32132707 10.1038/s41586-020-2078-2 PMC7147832

[138] Fessler E, Eckl EM, Schmitt S, Mancilla IA, Meyer-Bender MF, Hanf M, Philippou-Massier J, Krebs S, Zischka H, Jae LT (2020) A pathway coordinated by DELE1 relays mitochondrial stress to the cytosol. Nature 579:433–437. 10.1038/s41586-020-2076-4 32132706 10.1038/s41586-020-2076-4 PMC7116715

[139] Bi PY, Killackey SA, Schweizer L, Arnoult D, Philpott DJ, Girardin SE (2024) Cytosolic retention of HtrA2 during mitochondrial protein import stress triggers the DELE1-HRI pathway. Commun Biol. 10.1038/s42003-024-06107-7 38555279 10.1038/s42003-024-06107-7 PMC10981713

[140] Abdel-Nour M, Carneiro LAM, Downey J, Tsalikis J, Outlioua A, Prescott D, Da Costa LS, Hovingh ES, Farahvash A, Gaudet RG, Molinaro R, van Dalen R, Lau CCY, Azimi FC, Escalante NK, Trotman-Grant A, Lee JE, Gray-Owen SD, Divangahi M, Chen JJ, Philpott DJ, Arnoult D, Girardin SE (2019) The heme-regulated inhibitor is a cytosolic sensor of protein misfolding that controls innate immune signaling. Science. 10.1126/science.aaw4144 31273097 10.1126/science.aaw4144 PMC10433729

[141] Ron D, Walter P (2007) Signal integration in the endoplasmic reticulum unfolded protein response. Nat Rev Mol Cell Biol 8:519–529. 10.1038/nrm2199 17565364 10.1038/nrm2199

[142] Baleriola J, Walker CA, Jean YY, Crary JF, Troy CM, Nagy PL, Hengst U (2014) Axonally synthesized ATF4 transmits a neurodegenerative signal across brain regions. Cell 158:1159–1172. 10.1016/j.cell.2014.07.001 25171414 10.1016/j.cell.2014.07.001 PMC4149755

[143] Hanz S, Perlson E, Willis D, Zheng J-Q, Massarwa R, Huerta JJ, Koltzenburg M, Kohler M, van-Minnen J, Twiss JL, Fainzilber M (2003) Axoplasmic importins enable retrograde injury signaling in lesioned nerve. Neuron 40:1095–1104 14687545 10.1016/s0896-6273(03)00770-0

[144] Fessler E, Krumwiede L, Jae LT (2022) DELE1 tracks perturbed protein import and processing in human mitochondria. Nat Commun. 10.1038/s41467-022-29479-y 35388015 10.1038/s41467-022-29479-y PMC8986780

[145] Moisoi N, Klupsch K, Fedele V, East P, Sharma S, Renton A, Plun-Favreau H, Edwards RE, Teismann P, Esposti MD, Morrison AD, Wood NW, Downward J, Martins LM (2009) Mitochondrial dysfunction triggered by loss of HtrA2 results in the activation of a brain-specific transcriptional stress response. Cell Death Differ 16:449–464. 10.1038/cdd.2008.166 19023330 10.1038/cdd.2008.166

[146] Yun J, Cao JH, Dodson MW, Clark IE, Kapahi P, Chowdhury RB, Guo M (2008) Loss-of-function analysis suggests that Omi/HtrA2 is not an essential component of the pink1/parkin pathway *in vivo*. J Neurosci 28:14500–14510. 10.1523/JNEUROSCI.5141-08.2008 19118185 10.1523/JNEUROSCI.5141-08.2008 PMC2718055

[147] Tain LS, Chowdhury RB, Tao RN, Plun-Favreau H, Moisoi N, Martins LM, Downward J, Whitworth AJ, Tapon N (2009) *Drosophila* HtrA2 is dispensable for apoptosis but acts downstream of PINK1 independently from Parkin. Cell Death Differ 16:1118–1125. 10.1038/cdd.2009.23 19282869 10.1038/cdd.2009.23 PMC2711053

[148] Wang S, Long H, Hou L, Feng B, Ma Z, Wu Y, Zeng Y, Cai J, Zhang D, Zhao G (2023) The mitophagy pathway and its implications in human diseases. Signal Transduct Target Ther. 10.1038/s41392-023-01503-7 37582956 10.1038/s41392-023-01503-7 PMC10427715

[149] McWilliams TG, Prescott AR, Montava-Garriga L, Ball G, Singh F, Barini E, Muqit MMK, Brooks SP, Ganley IG (2018) Basal mitophagy occurs independently of PINK1 in mouse tissues of high metabolic demand. Cell Metab 27:439-449.e5. 10.1016/j.cmet.2017.12.008 29337137 10.1016/j.cmet.2017.12.008 PMC5807059

[150] Basak B, Holzbaur ELF (2025) Mitochondrial damage triggers the concerted degradation of negative regulators of neuronal autophagy. Nat Commun 16:7367. 10.1038/s41467-025-62379-5 40783388 10.1038/s41467-025-62379-5 PMC12335601

[151] Goldsmith J, Ordureau A, Harper JW, Holzbaur ELF (2022) Brain-derived autophagosome profiling reveals the engulfment of nucleoid-enriched mitochondrial fragments by basal autophagy in neurons. Neuron 110:967-976.e8. 10.1016/j.neuron.2021.12.029 35051374 10.1016/j.neuron.2021.12.029 PMC8930448

[152] Maday S, Holzbaur ELF (2016) Compartment-specific regulation of autophagy in primary neurons. J Neurosci 36:5933–5945. 10.1523/JNEUROSCI.4401-15.2016 27251616 10.1523/JNEUROSCI.4401-15.2016 PMC4887563

[153] Overly CC, Rieff HI, Hollenbeck PJ (1996) Organelle motility and metabolism in axons vs dendrites of cultured hippocampal neurons. J Cell Sci 109:971–980. 10.1242/jcs.109.5.971 8743944 10.1242/jcs.109.5.971

[154] Marahori NA, Gailer B, Schifferer M, Kleele T, Iatroudi A, Hannan SB, Avramopoulos P, Engelhardt S, Lakadamyali M, Brill MS, Misgeld T (2024) A retrograde transit filter mediated by optineurin controls mitostasis in distal axons. bioRxiv preprint. 10.1101/2024.07.28.604753

[155] Cai Q, Zakaria HM, Simone A, Sheng ZH (2012) Spatial parkin translocation and degradation of damaged mitochondria via mitophagy in live cortical neurons. Curr Biol 22:545–552. 10.1016/j.cub.2012.02.005 22342752 10.1016/j.cub.2012.02.005 PMC3313683

[156] Ordureau A, Kraus F, Zhang J, An H, Park S, Ahfeldt T, Paulo JA, Harper JW (2021) Temporal proteomics during neurogenesis reveals large-scale proteome and organelle remodeling via selective autophagy. Mol Cell 81:5082-5098.e11. 10.1016/j.molcel.2021.10.001 34699746 10.1016/j.molcel.2021.10.001 PMC8688335

[157] Lam WK, Lindblom RSJ, Milky B, Mazzachi P, Hadian-Jazi M, Küng C, Khuu G, Uoselis L, Nguyen TN, Skulsuppaisarn M, Saunders TL, Schmidt MF, Dewson G, Fogel AI, Bardy C, Lazarou M (2024) Presynapses are mitophagy pit stops that prevent axon degeneration. bioRxiv preprint. 10.1101/2024.09.09.611943

[158] Li Y, Zheng W, Lu Y, Zheng Y, Pan L, Wu X, Yuan Y, Shen Z, Ma S, Zhang X, Wu J, Chen Z, Zhang X (2022) BNIP3L/NIX-mediated mitophagy: molecular mechanisms and implications for human disease. Cell Death Dis 13. 10.1038/s41419-021-04469-y 10.1038/s41419-021-04469-y PMC8688453 34930907

[159] Valente EM, Abou-Sleiman PM, Caputo V, Muqit MMK, Harvey K, Gispert S, Ali Z, Del Turco D, Bentivoglio AR, Healy DG, Albanese A, Nussbaum R, González-Maldonado R, Deller T, Salvi S, Cortelli P, Gilks WP, Latchman DS, Harvey RJ, Dallapiccola B, Auburger G, Wood NW (2004) Hereditary early-onset Parkinson’s disease caused by mutations in PINK1. Science 304:1158–1160. 10.1126/science.1096284 15087508 10.1126/science.1096284

[160] Silvestri L, Caputo V, Bellacchio E, Atorino L, Dallapiccola B, Valente EM, Casari G (2005) Mitochondrial import and enzymatic activity of PINK1 mutants associated to recessive parkinsonism. Hum Mol Genet 14:3477–3492. 10.1093/hmg/ddi377 16207731 10.1093/hmg/ddi377

[161] Zhou C, Huang Y, Shao Y, May J, Prou D, Perier C, Dauer W, Schon EA, Przedborski S (2008) The kinase domain of mitochondrial PINK1 faces the cytoplasm. Proc Natl Acad Sci U S A 105:12022–12027. 10.1073/pnas.0802814105 18687899 10.1073/pnas.0802814105 PMC2575334

[162] Kato H, Lu Q, Rapaport D, Kozjak-Pavlovic V (2013) Tom70 is essential for PINK1 import into mitochondria. PLoS One. 10.1371/journal.pone.0058435 23472196 10.1371/journal.pone.0058435 PMC3589387

[163] Neupert W, Herrmann JM (2007) Translocation of proteins into mitochondria. Annu Rev Biochem 76:723–749. 10.1146/annurev.biochem.76.052705.163409 17263664 10.1146/annurev.biochem.76.052705.163409

[164] Meissner C, Lorenz H, Weihofen A, Selkoe DJ, Lemberg MK (2011) The mitochondrial intramembrane protease PARL cleaves human Pink1 to regulate Pink1 trafficking. J Neurochem 117:856–867. 10.1111/j.1471-4159.2011.07253.x 21426348 10.1111/j.1471-4159.2011.07253.x

[165] Greene AW, Grenier K, Aguileta MA, Muise S, Farazifard R, Haque ME, McBride HM, Park DS, Fon EA (2012) Mitochondrial processing peptidase regulates PINK1 processing, import and Parkin recruitment. EMBO Rep 13:378–385. 10.1038/embor.2012.14 22354088 10.1038/embor.2012.14 PMC3321149

[166] Yamano K, Youle RJ (2013) PINK1 is degraded through the N-end rule pathway. Autophagy 9:1758–1769. 10.4161/auto.24633 24121706 10.4161/auto.24633 PMC4028335

[167] Jin SM, Lazarou M, Wang C, Kane LA, Narendra DP, Youle RJ (2010) Mitochondrial membrane potential regulates PINK1 import and proteolytic destabilization by PARL. J Cell Biol 191:933–942. 10.1083/jcb.201008084 21115803 10.1083/jcb.201008084 PMC2995166

[168] Narendra D, Jin SM, Tanaka A, Suen D-F, Gautier CA, Shen J, Cookson MR, Youle RJ (2010) PINK1 is selectively stabilized on impaired mitochondria to activate Parkin. PLoS Biol. 10.1371/journal.pbio.1000298 20126261 10.1371/journal.pbio.1000298 PMC2811155

[169] Okatsu K, Oka T, Iguchi M, Imamura K, Kosako H, Tani N, Kimura M, Go E, Koyano F, Funayama M, Shiba-Fukushima K, Sato S, Shimizu H, Fukunaga Y, Taniguchi H, Komatsu M, Hattori N, Mihara K, Tanaka K, Matsuda N (2012) PINK1 autophosphorylation upon membrane potential dissipation is essential for Parkin recruitment to damaged mitochondria. Nat Commun. 10.1038/ncomms2016 22910362 10.1038/ncomms2016 PMC3432468

[170] Kane LA, Lazarou M, Fogel AI, Li Y, Yamano K, Sarraf SA, Banerjee S, Youle RJ (2014) PINK1 phosphorylates ubiquitin to activate parkin E3 ubiquitin ligase activity. J Cell Biol 205:143–153. 10.1083/jcb.201402104 24751536 10.1083/jcb.201402104 PMC4003245

[171] Kazlauskaite A, Kondapalli C, Gourlay R, Campbell DG, Ritorto MS, Hofmann K, Alessi DR, Knebel A, Trost M, Muqit MMK (2014) Parkin is activated by PINK1-dependent phosphorylation of ubiquitin at Ser65. Biochem J 460:127–139. 10.1042/BJ20140334 24660806 10.1042/BJ20140334 PMC4000136

[172] Koyano F, Okatsu K, Kosako H, Tamura Y, Go E, Kimura M, Kimura Y, Tsuchiya H, Yoshihara H, Hirokawa T, Endo T, Fon EA, Trempe JF, Saeki Y, Tanaka K, Matsuda N (2014) Ubiquitin is phosphorylated by PINK1 to activate parkin. Nature 510:162–166. 10.1038/nature13392 24784582 10.1038/nature13392

[173] Wang X, Winter D, Ashrafi G, Schlehe J, Wong YL, Selkoe D, Rice S, Steen J, Lavoie MJ, Schwarz TL (2011) PINK1 and Parkin target Miro for phosphorylation and degradation to arrest mitochondrial motility. Cell 147:893–906. 10.1016/j.cell.2011.10.018 22078885 10.1016/j.cell.2011.10.018 PMC3261796

[174] Narendra D, Tanaka A, Suen D-F, Youle RJ (2008) Parkin is recruited selectively to impaired mitochondria and promotes their autophagy. J Cell Biol 183:795–803. 10.1083/jcb.200809125 19029340 10.1083/jcb.200809125 PMC2592826

[175] Matsuda N, Sato S, Shiba K, Okatsu K, Saisho K, Gautier CA, Sou YS, Saiki S, Kawajiri S, Sato F, Kimura M, Komatsu M, Hattori N, Tanaka K (2010) PINK1 stabilized by mitochondrial depolarization recruits Parkin to damaged mitochondria and activates latent Parkin for mitophagy. J Cell Biol 189:211–221. 10.1083/jcb.200910140 20404107 10.1083/jcb.200910140 PMC2856912

[176] Sarraf SA, Raman M, Guarani-Pereira V, Sowa ME, Huttlin EL, Gygi SP, Harper JW (2013) Landscape of the PARKIN-dependent ubiquitylome in response to mitochondrial depolarization. Nature 496:372–376. 10.1038/nature12043 23503661 10.1038/nature12043 PMC3641819

[177] Ordureau A, Sarraf SA, Duda DM, Heo JM, Jedrychowski MP, Sviderskiy VO, Olszewski JL, Koerber JT, Xie T, Beausoleil SA, Wells JA, Gygi SP, Schulman BA, Harper JW (2014) Quantitative proteomics reveal a feedforward mechanism for mitochondrial PARKIN translocation and ubiquitin chain synthesis. Mol Cell 56:360–375. 10.1016/j.molcel.2014.09.007 25284222 10.1016/j.molcel.2014.09.007 PMC4254048

[178] Chen Y (1979) PINK1-phosphorylated mitofusin 2 is a Parkin receptor for culling damaged mitochondria. Science 340:471–475. 10.1126/science.1234493 10.1126/science.1231031 PMC3774525 23620051

[179] Tanaka A, Cleland MM, Xu S, Narendra DP, Suen DF, Karbowski M, Youle RJ (2010) Proteasome and p97 mediate mitophagy and degradation of mitofusins induced by Parkin. J Cell Biol 191:1367–1380. 10.1083/jcb.201007013 21173115 10.1083/jcb.201007013 PMC3010068

[180] McLelland G-L, Goiran T, Yi W, Dorval G, Chen CX, Lauinger ND, Krahn AI, Valimehr S, Rakovic A, Rouiller I, Durcan TM, Trempe J-F, Fon EA (2018) Mfn2 ubiquitination by PINK1/parkin gates the p97-dependent release of ER from mitochondria to drive mitophagy. Elife 7:e32866. 10.7554/eLife.32866.001 29676259 10.7554/eLife.32866 PMC5927771

[181] Lazarou M, Sliter DA, Kane LA, Sarraf SA, Wang C, Burman JL, Sideris DP, Fogel AI, Youle RJ (2015) The ubiquitin kinase PINK1 recruits autophagy receptors to induce mitophagy. Nature 524:309–314. 10.1038/nature14893 26266977 10.1038/nature14893 PMC5018156

[182] Stolz A, Ernst A, Dikic I (2014) Cargo recognition and trafficking in selective autophagy. Nat Cell Biol 16:495–501. 10.1038/ncb2979 24875736 10.1038/ncb2979

[183] Neuspiel M, Schauss AC, Braschi E, Zunino R, Rippstein P, Rachubinski RA, Andrade-Navarro MA, McBride HM (2008) Cargo-selected transport from the mitochondria to peroxisomes is mediated by vesicular carriers. Curr Biol 18:102–108. 10.1016/j.cub.2007.12.038 18207745 10.1016/j.cub.2007.12.038

[184] Soubannier V, Rippstein P, Kaufman BA, Shoubridge EA, McBride HM (2012) Reconstitution of mitochondria derived vesicle formation demonstrates selective enrichment of oxidized cargo. PLoS One. 10.1371/journal.pone.0052830 23300790 10.1371/journal.pone.0052830 PMC3530470

[185] Soubannier V, McLelland GL, Zunino R, Braschi E, Rippstein P, Fon EA, McBride HM (2012) A vesicular transport pathway shuttles cargo from mitochondria to lysosomes. Curr Biol 22:135–141. 10.1016/j.cub.2011.11.057 22226745 10.1016/j.cub.2011.11.057

[186] McLelland G-L, Soubannier V, Chen CX, McBride HM, Fon EA (2014) Parkin and PINK1 function in a vesicular trafficking pathway regulating mitochondrial quality control. EMBO J 33:282–295. 10.1002/embj.201385902 24446486 10.1002/embj.201385902 PMC3989637

[187] Lin MY, Cheng XT, Tammineni P, Xie Y, Zhou B, Cai Q, Sheng ZH (2017) Releasing syntaphilin removes stressed mitochondria from axons independent of mitophagy under pathophysiological conditions. Neuron 94:595-610.e6. 10.1016/j.neuron.2017.04.004 28472658 10.1016/j.neuron.2017.04.004 PMC5484086

[188] Herrup K, Yang Y (2007) Cell cycle regulation in the postmitotic neuron: oxymoron or new biology? Nat Rev Neurosci 8:368–378. 10.1038/nrn2124 17453017 10.1038/nrn2124

[189] Riddle D, Lichtenwalner R (2007) Neurogenesis in the Adult and Aging Brain. In: Riddle D (ed) Brain Aging: Models, Methods, and Mechanisms. CRC Press/Taylor & Francis, Boca Raton (FL) 21204350

[190] Desai R, East DA, Hardy L, Faccenda D, Rigon M, Crosby J, Soledad Alvarez M, Singh A, Mainenti M, Kuhlman Hussey L, Bentham R, Szabadkai G, Zappulli V, Dhoot GK, Romano LE, Xia D, Coppens I, Hamacher-Brady A, Paul Chapple J, Abeti R, Fleck RA, Vizcay-Barrena G, Smith K, Campanella M (2020) Mitochondria form contact sites with the nucleus to couple prosurvival retrograde response. Sci Adv 6:9955–9973. 10.1126/sciadv.abc9955 10.1126/sciadv.abc9955 PMC11206220 33355129

